# Effects of latroeggtoxin-VI on dopamine and α-synuclein in PC12 cells and the implications for Parkinson’s disease

**DOI:** 10.1186/s40659-024-00489-y

**Published:** 2024-03-16

**Authors:** Dianmei Yu, Haiyan Wang, Yiwen Zhai, Zhixiang Lei, Minglu Sun, Si Chen, Panfeng Yin, Xianchun Wang

**Affiliations:** State Key Laboratory of Developmental Biology of Freshwater Fish, Protein Chemistry Laboratory, College of Life Sciences, Hunan Normal University, Changsha, Hunan 410081 China

**Keywords:** Latroeggtoxin-VI, α-Synuclein, Dopamine, PC12 cell, PD mouse model, *L. tredecimguttatus*

## Abstract

**Background:**

Parkinson’s disease (PD) is characterized by death of dopaminergic neurons leading to dopamine deficiency, excessive α-synuclein facilitating Lewy body formation, etc. Latroeggtoxin-VI (LETX-VI), a proteinaceous neurotoxin discovered from the eggs of spider *L. tredecimguttatus*, was previously found to promote the synthesis and release of PC12 cells, showing a great potential as a drug candidate for PD. However, the relevant mechanisms have not been understood completely. The present study explored the mechanism underlying the effects of LETX-VI on dopamine and α-synuclein of PC12 cells and the implications for PD.

**Results:**

After PC12 cells were treated with LETX-VI, the level of dopamine was significantly increased in a dose-dependent way within a certain range of concentrations. Further mechanism analysis showed that LETX-VI upregulated the expression of tyrosine hydroxylase (TH) and L-dopa decarboxylase to enhance the biosynthesis of dopamine, and downregulated that of monoamine oxidase B to reduce the degradation of dopamine. At the same time, LETX-VI promoted the transport and release of dopamine through modulating the abundance and/or posttranslational modification of vesicular monoamine transporter 2 (VMAT2) and dopamine transporter (DAT). While the level of dopamine was increased by LETX-VI treatment, α-synuclein content was reduced by the spider toxin. α-Synuclein overexpression significantly decreased the dopamine level and LETX-VI efficiently alleviated the inhibitory action of excessive α-synuclein on dopamine. In the MPTP-induced mouse model of PD, application of LETX-VI ameliorated parkinsonian behaviors of the mice, and reduced the magnitude of MPTP-induced α-synuclein upregulation and TH downregulation. In addition, LETX-VI displayed neuroprotective effects by inhibiting MPTP-induced decrease in the numbers of TH-positive and Nissl-stained neurons in mouse brain tissues.

**Conclusions:**

All the results demonstrate that LETX-VI promotes the synthesis and release of dopamine in PC12 cells via multiple mechanisms including preventing abnormal α-synuclein accumulation, showing implications in the prevention and treatment of PD.

**Supplementary Information:**

The online version contains supplementary material available at 10.1186/s40659-024-00489-y.

## Background

Parkinson’s disease (PD) is recognised as the most common neurodegenerative disorder after Alzheimer’s disease [1] and is characterised by Lewy body formation, selective lose of dopaminergic neurons in the substantia nigra, dopamine deficiency, motor dysfunctions, etc. [1–3]. Loss of dopaminergic neurons within the substantia nigra is a crucial pathological feature of PD. It has been shown that moderate to severe dopaminergic neuronal loss within substantia nigra is probably the cause of motor dysfunctions [4]. Aggregation of abnormally folded α-synuclein to form Lewy bodies is another hallmark of PD. Aggregated abnormally folded α-synuclein is toxic to neurons and therefore Lewy bodies have a causal role in neuronal loss and dopamine deficiency [2, 5]. The main management strategy for PD involves the use of drugs that increase dopamine level in the brain or stimulate dopamine receptors, or use of anticholinergic drugs [2, 6]. The dopamine level in the brain can be increased by L-dopa, monoamine oxidase type B inhibitors, catechol-O-methyltransferase (COMT) inhibitors, etc. [7, 8]. Dopamine can not be used in the treatment of PD due to its inability to cross the blood-brain barrier (BBB). L-dopa is the precursor of dopamine and is able to cross the BBB. Therefore, L-dopa is often used in the treatment of PD [9]. However, the use of drugs to treat PD is often associated with serious side effects. For example, both L-dopa and dopamine agonists are associated with nausea, daytime somnolence and oedema; these side effects are more frequently observed when dopamine agonists are used [2]. Moreover, over time the patients with PD usually require more frequent administration of L-dopa, along with higher doses. Therefore, there is an urgent need to develop more targeted and efficient drugs to prevent or mitigate the degeneration of dopaminergic neurons, such as utilization of natural components to inhibit the apoptotic pathways in dopaminergic neurons [10] and protect dopaminergic neurons from the degeneration [11, 12]. The development of new drugs is expected to overcome the limitations of existing treatments and provide patients with more effective and sustainable treatment options.

Latroeggtoxin-VI (LETX-VI) is a proteinaceous neurotoxin discovered from the eggs of spider *L. tredecimguttatus.* Previous studies showed that LETX-VI promoted the synthesis and release of dopamine of PC12 cells, showing potential applications in the treatment of dopamine deficiency-related diseases, such as PD and depression [13–15]. However, the underlying mechanisms have not been clear completely. Our present study explored the effects of LETX-VI on dopamine and α-synuclein of PC12 cells and the implications for PD. LETX-VI was found to promote dopamine synthesis and release via multiple mechanisms and prohibit abnormal α-synuclein accumulation, showing implications in the prevention and treatment of PD via endogenous pathways.

## Results

### Effects of LETX-VI on the levels of dopamine and α-synuclein

**Fig. 1:**
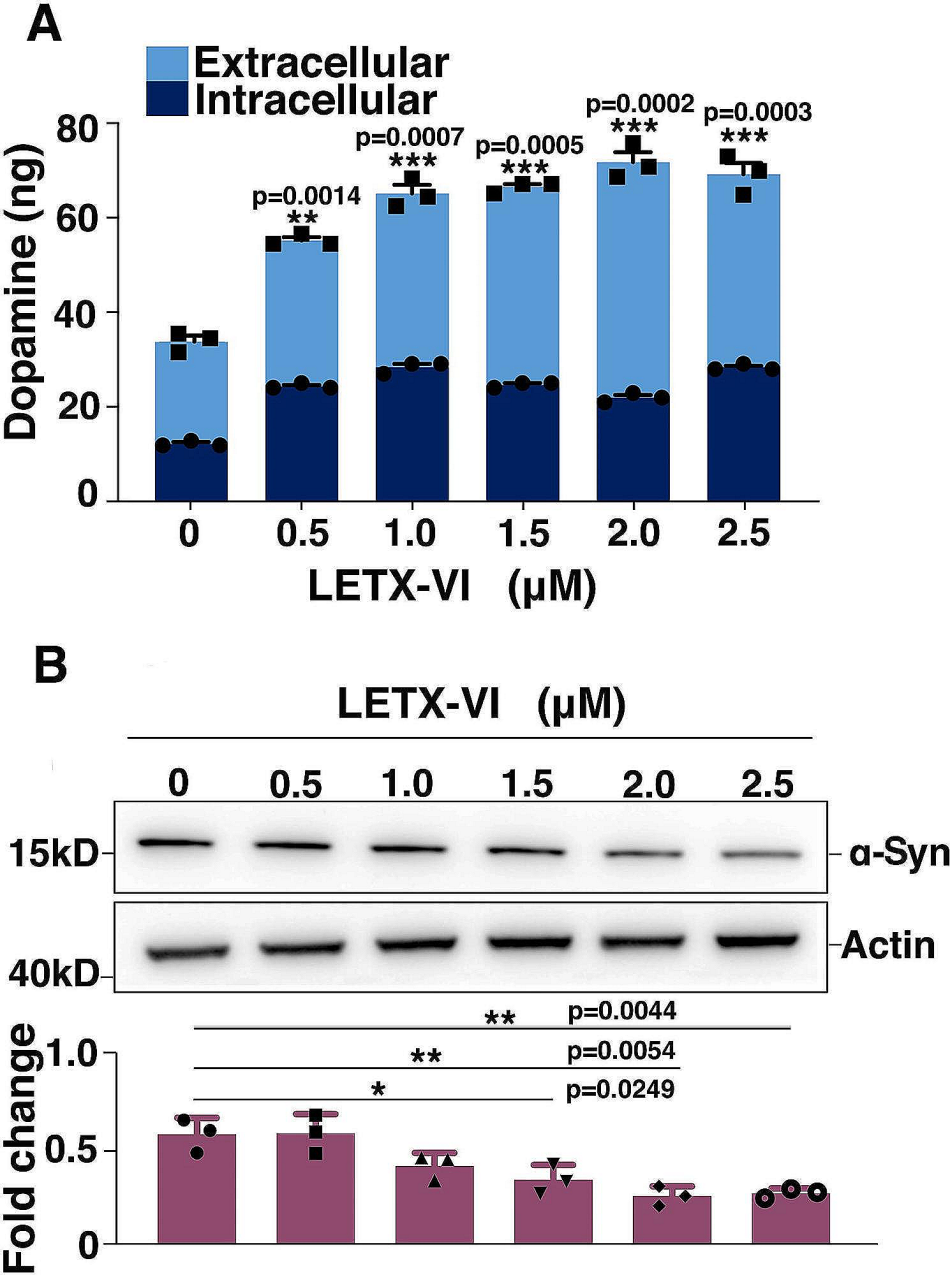
Effects of LETX-VI on the levels of dopamine and α-synuclein. **A**: Effect LETX-VI at different concentrations on the level of dopamine. **B**: Effect LETX-VI at different concentrations on the level of α-synuclein (α-Syn). *P **<** 0.05, **P **<** 0.01, * **P < 0.001 vs. control. *n* ≥ 3

In view of the fact that both dopamine and α-synuclein are related with PD, we detected the effects of LETX-VI at different concentrations (0, 0.5, 1, 1.5, 2, 2.5 µM) on the levels of dopamine and α-synuclein under the same experimental conditions. As shown in Fig. 1A, treatment of PC12 cells with LETX-VI in the concentration range of 0.5–2.5 µM led to an increase in the total and released amounts of dopamine (*P* < 0.01 or 0.001), suggesting that LETX-VI has the potential to promote the synthesis and excretion of dopamine of PC12 cells. Western blot analysis confirmed that the level of α-synuclein was decreased with increasing LETX-VI concentrations up to 2.5 µM (Fig. 1B) (*P* < 0.05 or 0.01), indicating that dopamine and α-synuclein showed opposite change tendencies during LETX-VI treatment.

### α-synuclein overexpression and its influence on dopamine

In view of the above results, the α-synuclein decrease was speculated to have a relationship with the dopamine increase during LETX-VI treatment. For confirming this speculation, the influence of α-synuclein overexpression on dopamine was analyzed. We constructed the expression vector containing the gene *SNCA* encoding rat α-synuclein using pcDNA 3.1(+). After the resultant expression plasmid pcDNA 3.1(+)-*SNCA* was digested with BamHI/EcoRI, agarose gel electrophoresis indicated that there were two bands appearing, with sizes about 5400 bp and 400 bp, respectively (Additional file 1 A), which were consistent with the theoretical values of vector and desired product, respectively, demonstrating that the expression plasmid was constructed successfully. Western blot analysis confirmed that, when 3 µl Lipofectamin 2000 and 3 µg pcDNA 3.1 (+) - *SNCA* (ratio 1:1) were applied, the expression of α-synuclein was the highest (Additional file 1B) (*P* < 0.01), which was used as the optimal transfection conditions in the following overexpression experiments.

**Fig. 2:**
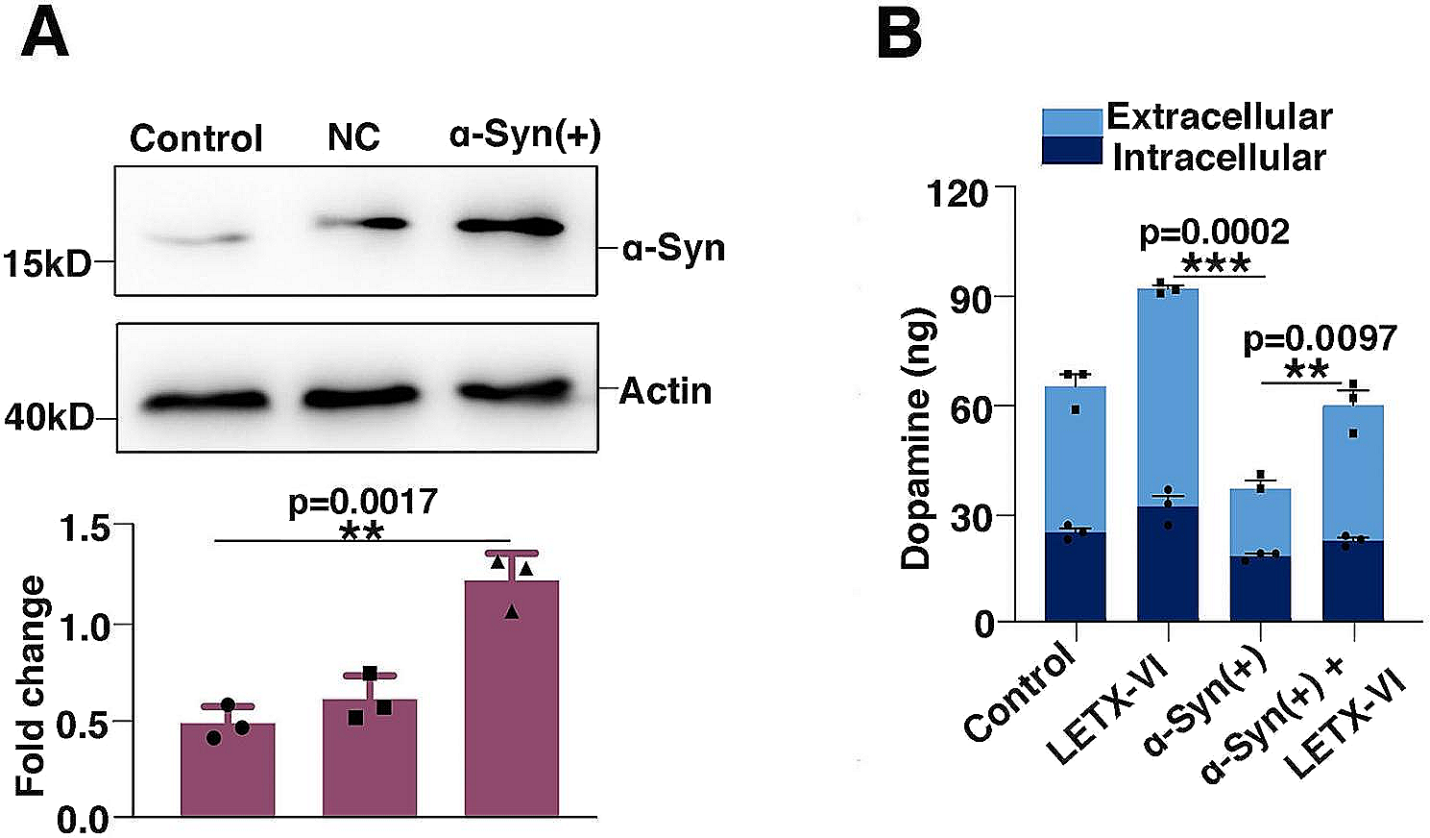
α-Synuclein overexpression and its influence on dopamine. **A**: Western blot analysis of the efficiency of α-synuclein overexpression. **B**: Effects of LETX-VI and α-synuclein overexpression on dopamine level of PC12 cells. LETX-VI: treatment of wild-type PC12 cells by LETX-VI. α-Syn (+): PC12 cells with overexpressed α-Syn. α-Syn (+) + LETX-VI: treatment of PC12 cells with overexpressed α-synuclein by LETX-VI. **P **<** 0.01, * **P < 0.001. *n* ≥ 3

When the overexpression of α-synuclein was performed under the optimized experimental conditions, western blot analysis showed that the level of α-synuclein in the PC12 cells was significantly increased compared with the control as well as the negative control (*P* < 0.01), demonstrating that the overexpression was successful (Fig. 2A). Figure 2B shows that, compared with the control, LETX-VI treatment of PC12 cells resulted in an increase in the amount of dopamine; α-synuclein overexpression led to a remarkable decrease in the levels of both extracellular and intracellular dopamine (*P* < 0.001); however, the application of LETX-VI to the PC12 cells with the gene *SNCA* being overexpressed significantly mitigated the adverse influence of α-synuclein overexpression on dopamine (*P* < 0.01). These results demonstrate that excessive α-synuclein decreases dopamine level, and LETX-VI can increase dopamine by reducing the expression level of α-synuclein.

### LETX-VI entered the nucleus of PC12 cells to promote tyrosine hydroxylase expression

**Fig. 3:**
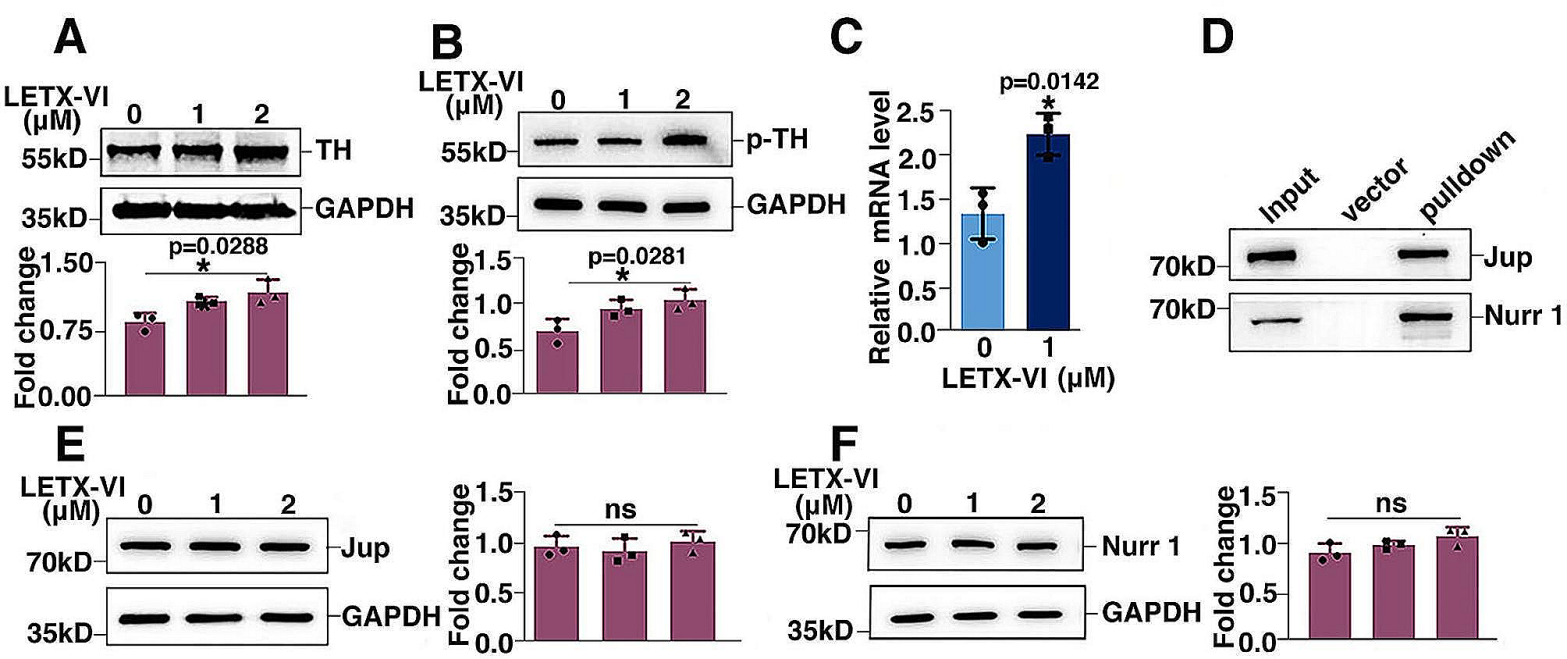
LETX-VI entered the nucleus of PC12 cells to promote tyrosine hydroxylase (TH) expression. **A**: LETX-VI increased TH abundance. **B**: LETX-VI up-regulated the level of phosphorylated TH at S31(p-TH). **C**: LETX-VI promoted transcription of TH confirmed by qPCR. *P < 0.05 vs. control. **D**: LETX-VI interacted with junction plakoglobin (Jup) and nuclear receptor related protein 1 (Nurr 1) verified with pulldown combined with western blot analysis. Input: PC12 cell lysate. Vector: showing that the nickel beads for fixing LETX-VI did not adsorb Jup and Nurr1. Pulldown: proteins pulled down from PC12 cell lysate by LETX-VI. **E**: LETX-VI had no obvious effect on the level of junction Jup. **F**: LETX-VI had no obvious effect on the level of Nurr 1. *P **<** 0.05. *n* ≥ 3

Tyrosine hydroxylase (TH) is the rate-limiting enzyme for dopamine biosynthesis. When we probed into the mechanism for LETX-VI to increase dopamine level, this enzyme was first paid attention to. Western blot analysis indicated that treatment of PC12 cells with 1.0 µM and 2.0 µM LETX-VI for 24 h led to an increase in the level of TH in a dose-dependent manner (*P* < 0.05) (Fig. 3A). Besides, we used anti-phosphorylated TH (Phospho S31) antibody to detect the phosphorylation of TH, and found that 2.0 µM LETX-VI increased the phosphorylation level of the enzyme at S31 (*P* < 0.05) (Fig. 3B), which is favorable for regulating TH subcellular localization and increasing the dopamine synthesis [16]. These results suggest that LETX-VI promotes the biosynthesis of dopamine by upregulating TH expression and its phosphorylation level. In addition, we also quantitatively determined the mRNA for TH with qPCR, and the results shown in Fig. 3C indicate that 1.0 µM LETX-VI treatment for 24 h significantly increased the level of the mRNA for TH (*P* < 0.05). These observations demonstrate that LETX-VI upregulates the expression of TH at both transcription and translation levels.

For further revealing the mechanism of action of LETX-VI in promoting TH expression, we detected the possible interaction of LETX-VI with junction plakoglobin and nuclear receptor related protein 1 using HIS-tagged LETX-VI (HIS-LETX-VI) pulldown combined with western blot analysis. Junction plakoglobin (also known as plakoglobin or gamma-catenin) positively regulates protein import into the nucleus [17, 18] and our previous work using mass spectrometry-based methods identified that junction plakoglobin is one of the interacting proteins of LETX-VI (Data not shown). Nuclear receptor related protein 1 has been reported to promote the transcription of TH gene in murine and rat [19]. The results (Fig. 3D) showed that LETX-VI could pull down junction plakoglobin and nuclear receptor related protein 1 from the PC12 cell lysate, indicating that there is an interaction between LETX-VI and these two proteins. These results suggest that LETX-VI can enter the nucleus of PC12 cells with the help of junction plakoglobin and then promote the transcription of the gene encoding TH by interacting with the transcription factor nuclear receptor related protein 1, which is supported by the previous report that LETX-VI is able to enter the nucleus confirmed by fluorescent labeling experiments [13]. In addition, treatment of PC12 cells with LETX-VI did not influence the intracellular levels of junction plakoglobin (Fig. 3E) and nuclear receptor related protein 1 (Fig. 3F), demonstrating that LETX-VI promoted the expression of TH by interacting with the existing junction plakoglobin and nuclear receptor related protein 1, without changing their levels.

### Effects of LETX-VI on other several enzymes closely related to dopamine metabolism

**Fig. 4:**
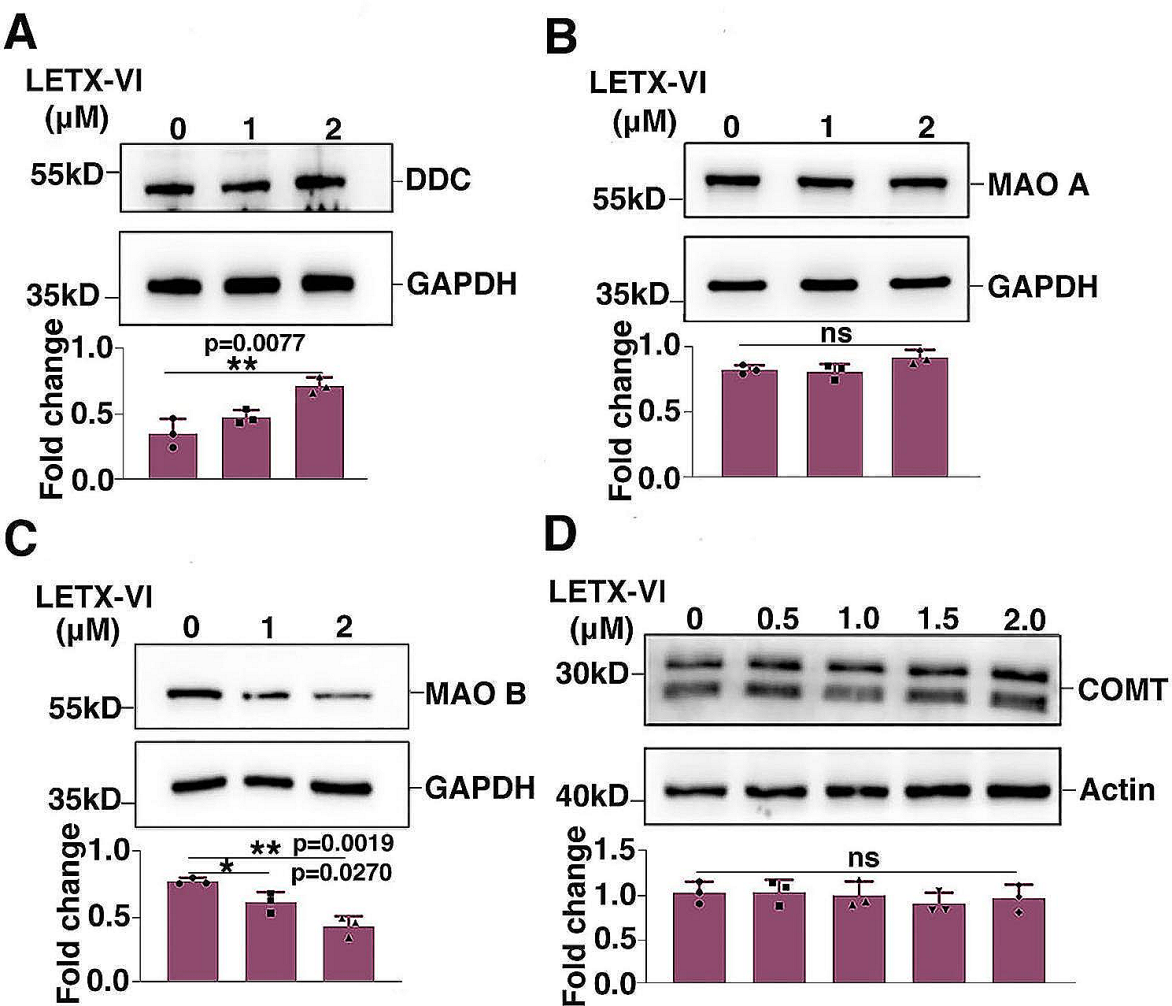
Western blot analysis of the effects of LETX-VI on the levels of L-dopa decarboxylase, monoamine oxidases and catechol-O-methyltransferase. DDC: L-dopa decarboxylase. MAO A: monoamine oxidase (A) MAO B: monoamine oxidase (B) COMT: catechol-O-methyltransferase. **P < 0.01, *P < 0.05. *n* ≥ 3

After tyrosine was converted by TH to L-dihydroxyphenylalanine (L-dopa), the latter was in turn converted by L-dopa decarboxylase to dopamine, followed by storage in vesicles and release via exocytosis. The action of released dopamine is terminated by re-absorption into the cell or degradation to homovanillic acid and normetanephrine for further metabolism or excretion, catalyzed by monoamine oxidases, catechol-O-methyltransferase and dopamine β-hydroxylase [20]. In PC12 cells, there are no epinephrine produced and the content of norepinephrine is low [21, 22]; thus the degradation of dopamine in PC12 cells is mainly controlled by monoamine oxidases and catechol-O-methyltransferase. Therefore, we detected the effects of LETX-VI on L-dopa decarboxylase, monoamine oxidase A, monoamine oxidase B, and catechol-O-methyltransferase. Western blot analysis confirmed that after treatment of PC12 cells with LETX-VI at up to 2.0 µM for 24 h, the content of L-dopa decarboxylase was increased (Fig. 4A) (*P* < 0.01), whereas that of monoamine oxidase B was deceased (Fig. 4C) (*P* < 0.05 or 0.01), with the contents of monoamine oxidase A and catechol-O-methyltransferase being not obviously altered (Fig. 4B, D). These results suggest that LETX-VI increases the total amount of dopamine of PC12 cells by up-regulating TH and L-dopa decarboxylase to promote dopamine biosynthesis, and down-regulating monoamine oxidase B to reduce dopamine degradation.

### Effects of LETX-VI on VMAT2 and DAT

**Fig. 5:**
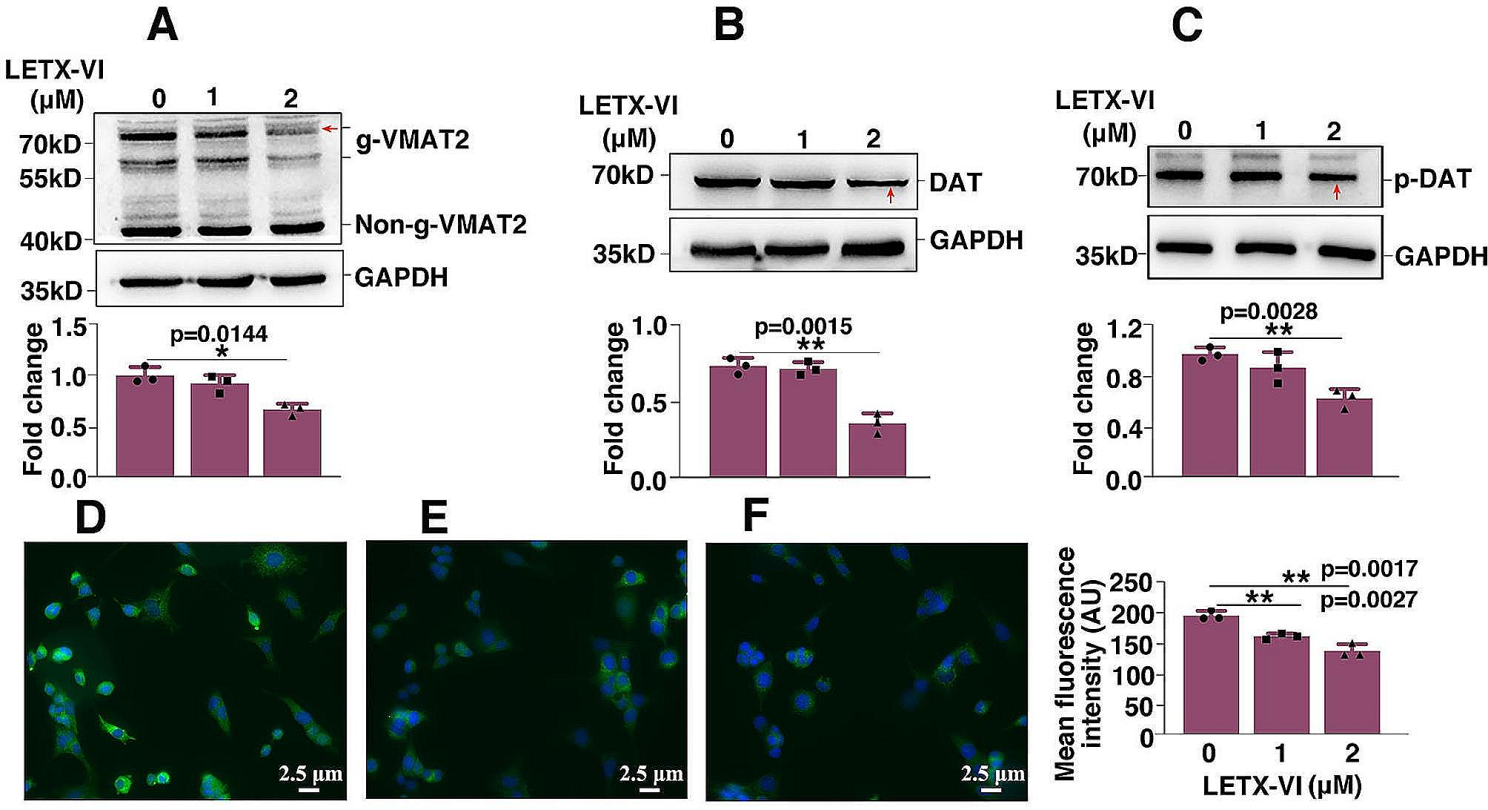
Effects of LETX-VI on VMAT2 and DAT. **A**: Effect of LETX-VI on the contents of glycosylated vesicular monoamine transporter 2 (g-VMAT2) and non-glycolslyated vesicular monoamine transporter 2 (non-g-VMAT2). **B**: Effect of LETX-VI on the content of dopamine transporter (DAT). **C**: Effect of LETX-VI on the content of dopamine transporter phosphorylated at T53 (p-DAT). **D**: Content of DAT detected with fluorescent-labeled antibody before LETX-VI treatment. **E**: Content of DAT detected with fluorescent-labeled antibody after 1 µM LETX-VI treatment for 24 h. **F**: Content of DAT detected with fluorescent-labeled antibody after 2 µM LETX-VI treatment for 24 h. The 4× and 10× immunofluorescence images for DAT analysis were shown in the the Additional file 2: Supplementary 4× and 10× immunofluorescence and Nissl staining images. *P < 0.05, ** *P* < 0.01. *n* ≥ 3

To further understand the effects of LETX-VI on localization and release of dopamine, the LETX-VI caused-changes in the contents of vesicular monoamine transporter 2 (VMAT2) and dopamine transporter (DAT) were also analyzed. As shown in Fig. 5A, LETX-VI treatment of PC12 cells gave rise to a decrease in the content of glycosylated VMAT2 (*P* < 0.05), with the content of non-glysosylated VMAT2 unchanged, suggesting that LETX-VI treatment influences the distribution of VMAT2 between different types of secretory vesicles. In contrast, the contents of DAT and phosphorylated DAT at T53 were decreased by LETX-VI in a dose-dependent manner (Fig. 5B, C) (*P* < 0.01), suggesting that the reuptake of dopamine by DAT from outside the cells was decreased after LETX-VI treatment, which is favorable for enhancing the action of released dopamine (see Discussion). For further verifying the LETX-VI-caused decrease in DAT content, we employed fluorescent-labeled antibody to detect the changes in the content of DAT after treatment with LETX-VI at 1 and 2 µM for 24 h, respectively. The results (Fig. 5D- F) indicated that, compared with the control, the mean fluorescent intensity within PC12 cells was decreased with increasing the LETX-VI concentrations, further confirming that LETX-VI treatment led to a decrease in the content of DAT (*P* < 0.01) in the cells. All the observations demonstrate that LETX-VI can modulate the transfer of dopamine by changing the levels and/or post-translational modification of VMAT2 and DAT proteins.

### LETX-VI ameliorated the behavior deficit of PD model mice

**Fig. 6:**
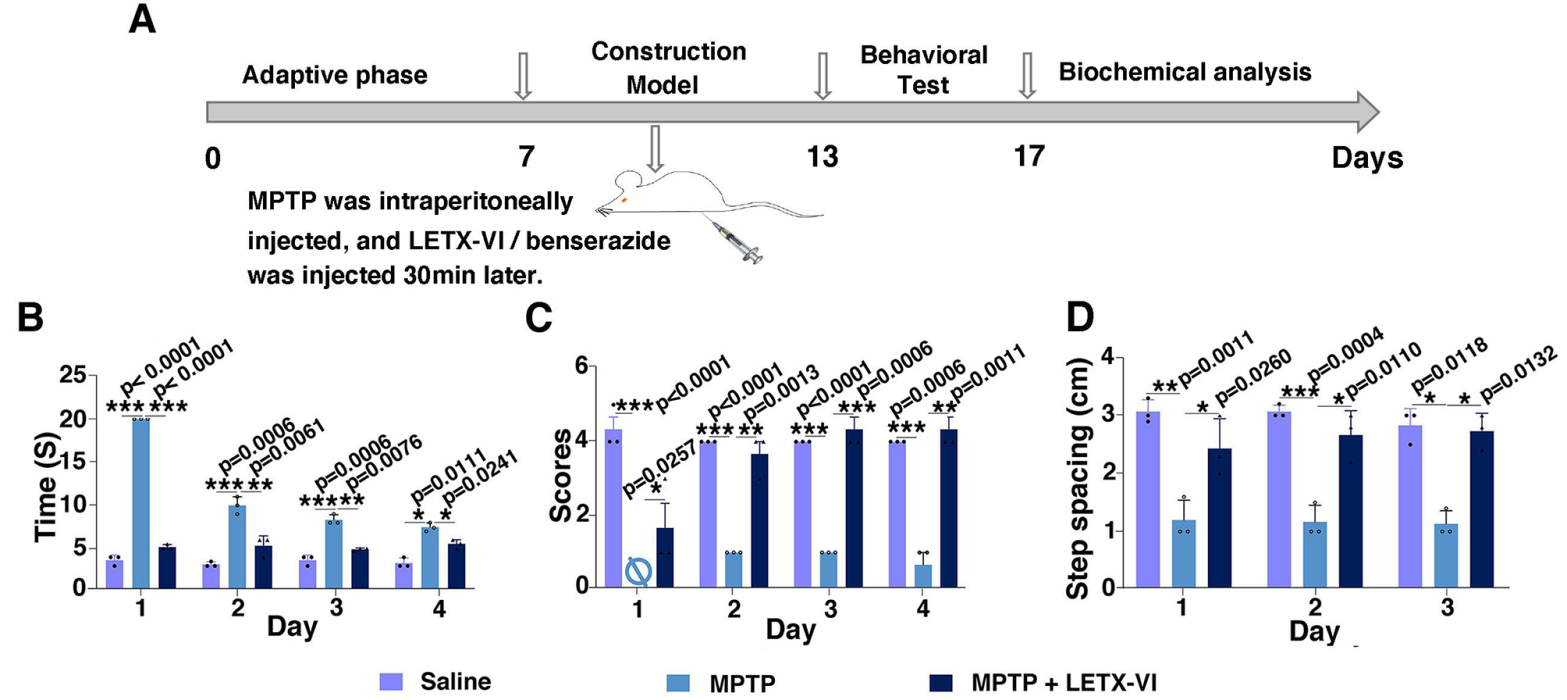
LETX-VI ameliorated the behavior deficit of PD model mice. **A**: The scheme of the experiments. **B**: Effects of MPTP and LETX-VI on the pole-climbing ability of the PD model mice during 4 days after the 6-day drug administration. **C**: Effects of MPTP and LETX-VI on the wire-hanging ability of the PD model mice during 4 days after the 6-day drug administration. **D**: Effects of MPTP and LETX-VI on the step spacing of the PD model mice during 3 days after the 6-day drug administration. * *P* < 0.05, **P < 0.01, * **P < 0.001. *n* ≥ 3

In view of the above results that LETX-VI could prevent abnormal accumulation of α-synuclein and promote the synthesis and secretion of dopamine, LETX-VI was suggested to be able to combat PD. Hence, we developed a PD mouse model with MPTP to evaluate the antiparkinsonian effects of LETX-VI. The scheme of the relevant experiments is shown in Fig. 6A. Figure 6B shows the effects of MPTP and LETX-VI on the pole-climbing time of mice. During the 4 days after the completion of PD model development, pole-climbing time of model group mice was decreased as the day number increased (*P* < 0.05, 0.01 or 0.001), and LETX-VI obviously shortened the MPTP-lengthened pole-climbing time.

When wire-hanging test was performed within 4 days after completion of the model development, the score of model group mice in wire-hanging test was significantly decreased compared with the control (*P* < 0.01 or 0.001), with zero point on the first day. When MPTP was applied in combination with LETX-VI, the MPTP-shortened wire-hanging time on days 2–4 was significantly lengthened by LETX-VI (Fig. 6C). The results of footprint test (Fig. 6D) showed that during the 3 days after model development, the step spacing of the mice in model group was found to be significantly decreased by MPTP (*P* < 0.05 or 0.01 or 0.001), and LETX-VI attenuated the MPTP-induced decrease in the step spacing. All the observations demonstrate that LETX-VI significantly ameliorates MPTP-induced behavioral impairments of PD model mice.

### In vivo levels of selected proteins closely related to dopamine and/or PD

**Fig. 7:**
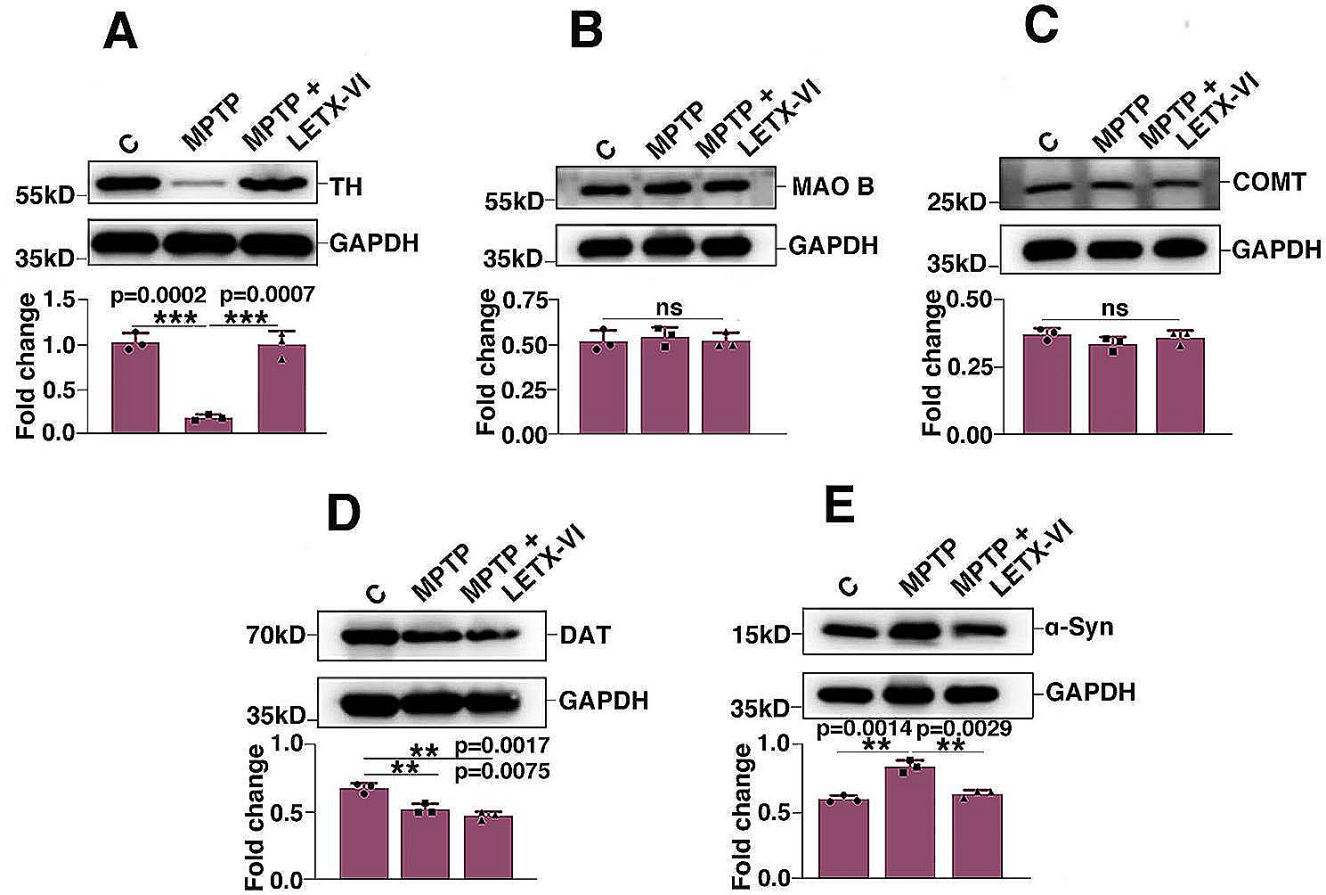
Effects of MPTP and LETX-VI on the levels of selected proteins in brain tissues of PD model mice. **A**: Effects of MPTP and LETX-VI on the level of tyrosine hydroxylase (TH). **B**: Effects of MPTP and LETX-VI on the level of monoamine oxidase B (MAO B). **C**: Effects of MPTP and LETX-VI on the level of catechol-O-methyltransferase (COMT). **D**: Effects of MPTP and LETX-VI on the level of dopamine transporter (DAT). **E**: Effects of MPTP and LETX-VI on the level of α-synuclein (α-Syn). *P < 0.05, **P < 0.01, * **P < 0.001. *n* ≥ 3

In view of the importance of a series of proteins, including TH and α-synuclein, in dopamine metabolism and/or PD development, their levels in the nigrostriatal tissues of PD model mice were also determined after behavioral tests. The in vivo and in vitro detection results were compared so as to further confirm the anti-PD effects of LETX-VI. As shown in Fig. 7A, MPTP treatment led to a remarkable reduction in the abundance of TH compared with the control (*P* < 0.001), and however when MPTP was applied in combination with LETX-VI, the abundance of TH was not obviously decreased, indicating that LETX-VI is able to inhibit MPTP-induced decrease in the content of TH. Such an in vivo promoting effect of LETX-VI on TH level is consistent with that of the in vitro experiments (Fig. 3). Figure 7B and C show that application MPTP alone or in combination with LETX-VI did not obviously influence the levels of monoamine oxidase B and catechol-O-methyltransferase, although in vitro experiments LETX-VI was shown to decrease the content of monoamine oxidase B (Fig. 4). MPTP alone decreased the content of DAT and the combined application of MPTP and LETX-VI further decreased the content (*P* < 0.01), showing that LETX-VI, like the situation in the in vitro experiments (Fig. 4), has a suppressive effect on the DAT level (Fig. 7D). MPTP led to an increase in the abundance of α-synuclein, which is consistent with the report in literature [23]. LETX-VI inhibited the MPTP-induced up-regulation of α-synuclein (Fig. 7E) (*P* < 0.01) and the in vivo observation about the effect of LETX-VI on α-synuclein is in agreement with that of the in vitro experiments (Fig. 1B).

### Tyrosine hydroxylase immunofluorescence and Nissl staining

**Fig. 8:**
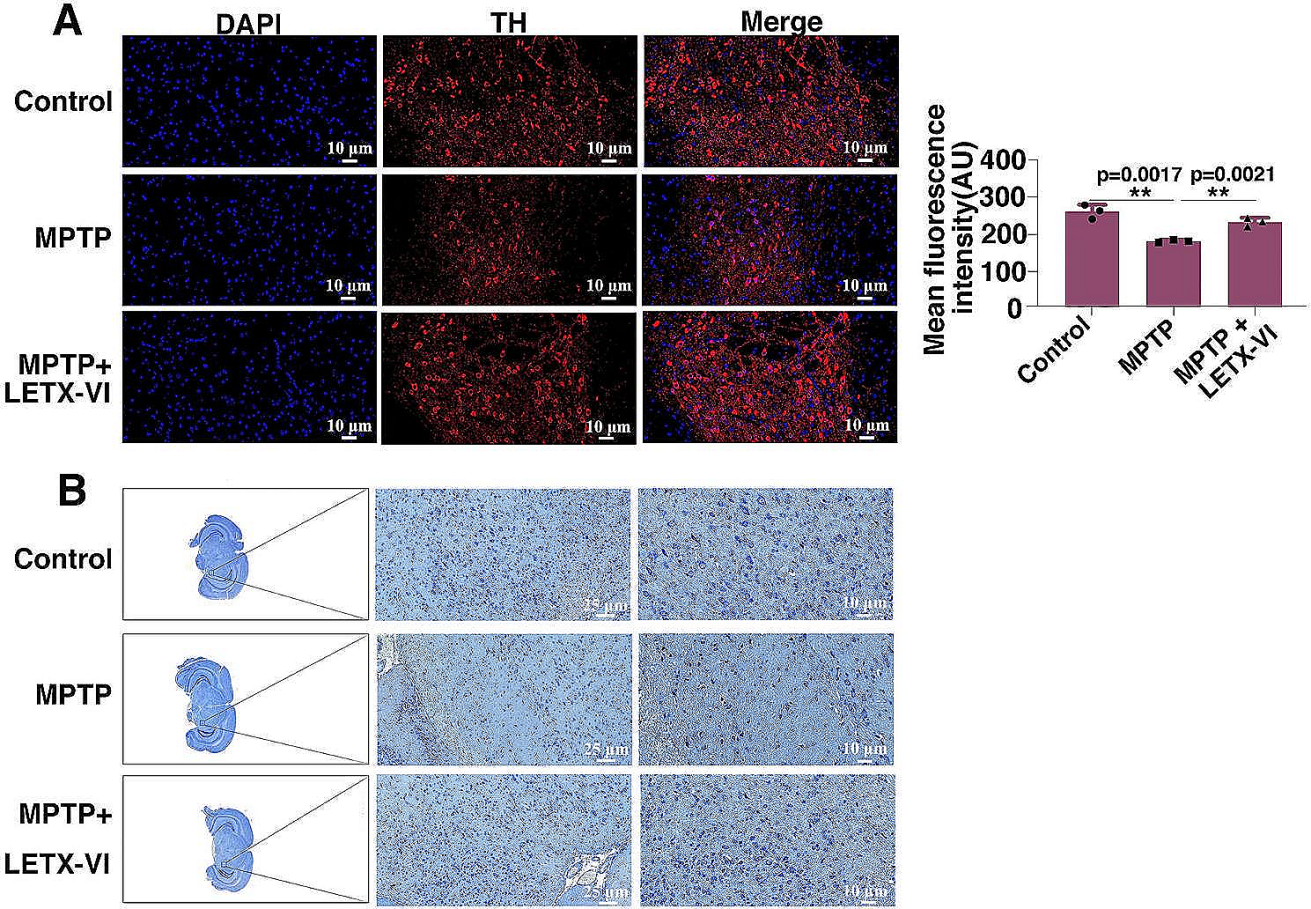
Tyrosine hydroxylase (TH) immunofluorescence and Nissl staining of the nigrostriatal region of the mice. **A**: Nigrostriatal TH immunofluorescence. **B**: Nissl staining of brain tissues, showing the enlarged nigrostriatal region. The 4× and 10× immunofluorescence and Nissl staining images were shown in the Additional file 2: Supplementary 4× and 10× immunofluorescence and Nissl staining images. MPTP: PD model mice induced with MPTP. MPTP + LETX-VI: LETX-VI was injected 30 min after MPTP administration. DAPI: 4’,6-diamidino-2-phenylindole. TH: tyrosine hydroxylase. *P < 0.05, ** *P* < 0.01. *n* ≥ 3

In order to investigate the potential neuroprotective effect of LETX-VI against MPTP toxicity, TH immunofluorescence and Nissl staining analyses of the nigrostriatal region of the mice were performed. As shown in Fig. 8A, compared with the control, MPTP treatment led to a remarkable decrease in the number and mean fluorescence intensity of the TH-positive neurons in the nigrostriatal region (*P* < 0.01), and application of LETX-VI after MPTP administration protected TH-positive neurons from the toxicity of MPTP, causing the number and mean fluorescence intensity of the TH-positive neurons to be comparable to those of the control (Fig. 8A, right), which were quantitatively analyzed with ImageJ software (https://imagej.en.softonic.com). Nissl bodies are important sites for protein synthesis and more Nissl bodies means more living neurons [24, 25]. When Nissl staining of the nigrostriatal region was performed, MPTP was found to result in a significant loss of Nissl-stained neurons in the nigrostriatal region; however, when MPTP was given in combination with LETX-VI, the number of Nissl-stained neurons was not obviously different from that of the control (Fig. 8B). These data suggest that LETX-VI is able to protect the neurons from the toxicity of MPTP in the PD model mice.

## Discussion

The PC12 cell line, derived from a rat pheochromocytoma, has the ability to synthesize, store and release neurotransmitters, including catecholamines. The metabolism of catecholamines in these cells is very similar to that in the sympathetic neurons requiring the same metabolic enzymes, and is regulated by similar second messenger systems in mammals. Therefore, PC12 cells become a neuronal cell model for studying the synthesis and release of neurotransmitters and are widely used to elucidate the mechanism of action of a series of neurotoxins and drugs [26–31]. In our present study, LETX-VI was found to enter the cytoplasm and nucleus of PC12 cells to modulate relevant gene expression and proteins/enzyme metabolism, affecting the level of dopamine in PC12 cells through multiple mechanisms. First of all, LETX-VI upregulated the expression of TH and L-dopa decarboxylase, and promoted the phosphorylation of TH at S31, thus not only increasing the contents of TH and L-dopa decarboxylase, but also enhancing the catalytic activity of TH [16]. At the same time, the level of monoamine oxidase B, an important enzyme degrading dopamine, was downregulated by LETX-VI. The decrease in monoamine oxidase B is favorable not only for reducing dopamine degradation, but also for mitigating the adverse influence of dopamine metabolites on α-synuclein protein. It is generally believed that dopamine metabolites in dopaminergic neurons promote the aggregation of toxic α-synuclein oligomers and protofibrils, whereas dopamine inhibits the formation of toxic fibers and even degrades the toxic fibers [32]. Monoamine oxidases are divided into two isoenzymes, monoamine oxidase A and monoamine oxidase B. Monoamine oxidase B preferentially acts in dopamine catabolism in humans and monoamine oxidase A is the primary monoamine oxidase for dopamine catabolism in the brain of mice [33]. Our experiments found that LETX-VI significantly decresed the level of monoamine oxidase B in PC12 cells (Fig. 4B), suggesting that LETX-VI may play an important role in the inhibition of dopamine degradation in humans. The inhibitors of monoamine oxidase B are used in the treatment of PD because they can increase synaptic dopamine by blocking its degradation [34]. LETX-VI was suggested to function in the treatment of PD like the inhibitors of monoamine oxidase B at least to a certain degree.

After dopamine is synthesized, its vesicular localization, release and extracellular persistence are controlled largely by the coordinated actions of VMAT2 and DAT [35]. VMAT2 transports dopamine from the cytosol into synaptic vesicles. Therefore, The activity of VMAT2 significantly influences the scale of neurotransmitter release [36, 37]. On the other hand, rapid transport of the synthesized dopamine into vesicles by VMAT2 prevents the accumulation of cytosolic dopamine that could be converted into neurotoxic species [38]. LETX-VI caused-downregulation of g-VMAT2 explains the promoting effect of LETX-VI on dopamine release and its neuroprotection effect from one aspect. Within the central nervous system (CNS), VMAT2 is primarily localized to the membrane of small synaptic vesicles, with only a small portion to that of dense-core vesicles [39]. N-linked glycosylation of the C terminus and the linking region between the first and second transmembrane domains of VMAT2 is responsible for its vesicular targeting. Glycosylation is necessary to traffic VMAT2 to dense-core vesicles, and in the absence of C-terminal and linking domain glycosylation, VMAT2 is trafficked to small vesicles [35, 40]. LETX-VI was found to downregulate the glycosylation level of VMAT2, suggesting that LETX-VI promotes VMAT2 trafficking to small vesicles. DAT is a phosphoprotein expressed in the plasma membrane and can be relocated into the interior of dopaminergic neurons [35]. In the CNS, level of extraneuronal dopamine is controlled primarily by the action of DAT in the plasma membrane [41]. Phosphorylation of DAT at T53 is involved in its transport activity, because mutation of T53 of DAT prevents phosphorylation and leads to a reduced dopamine transport Vmax [42]. Our present study found that LETX-VI decreased the levels of DAT and phosphorylated DAT at T53, demonstrating that the reuptake of dopamine was weakened by LETX-VI, which is helpful to enhance the action of secreted dopamine. These observations demonstrate that LETX-VI is able to modulate the transfer and release of dopamine by changing the abundance and/or posttranslational modification of VMAT2 and DAT proteins.

There are many natural venom components targeting the monoaminergic system at many different points, affecting the synthesis, transport, release and reuptake of monoamines, acting as agonists and antagonists at monoaminergic receptors, changing the sensitivity of receptors, etc. [43]. For example, huwentoxin-I, a peptide neurotoxin, significantly promotes dopamine release from PC12 cells [44]. The venom of the parasitoid wasp affects the dopamine receptors in normal cockroaches [45]. Melittin inhibits the function of DAT and thus affects dopamine availability [46]. Comparatively, LETX-VI is able to promote the synthesis, transport and release of dopamine through multiple mechanisms, thereby showing even better application prospects in the relevant fields.

After LETX-VI treatment, the levels of dopamine and α-synuclein in PC12 cells displayed opposite change tendencies: the level of dopamine was increased and that of α-synuclein was decreased, suggesting that there may be a negative correlation bwtween them. We used α-synuclein overepression experiment confirmed the relationship between α-synuclein and dopamine as well as the effects of LETX-VI on them. The results showed that excessive α-synuclein resulted in decreased dopamine, which was attributed to the cell injury caused by abnormal accumulation of α-synuclein [47–49]. LETX-VI treatment of the PC12 cells with α-synuclein overpexpression rescued the decreased dopamine level caused by excessive α-synuclein (Fig. 2). These observations indicate that preventing abmormal accumulation of α-synuclein and decreasing its injury toward neurons are one of the molecular machanisms for LETX-VI to increase the dopamine level. Multiple E3 ubiquitin-protein ligases, including E3 ubiquitin–protein ligase NEDD4, have been shown to ubiquitinate α-synuclein and promote its subsequent degradation via the proteasome or lysosome [50]. Our previous work had demonstrated that E3 ubiquitin–protein ligase NEDD4 is one of the interacting proteins of LETX-VI [14], suggesting that LETX-VI maybe decreases the level of α-synuclein in the PC12 cells via ubiquitination-proteasome degradation pathway, which needs further investigation. In addition, that LETX-VI treatment of PC12 cells prevented the abnormal accumulation of α-synuclein also suggests that LETX-VI could exert neuroprotectve action by avoiding or reducing neuron injury caused by excessive α-synuclein, which was supported by the in vivo expriments using the PD mouse model.

α-Synuclein is a 14 kDa protein with the ability to inhibit the activity of TH in a dose-dependent manner and thus adversely affects the synthesis of dopamine [51]. Overexpression of α-synuclein results in the inhibition of synaptic vesicle exocytosis; however, loss of α-synuclein does not have a major effect on synaptic transmission [52], which suggests that the α-synuclein decrease caused by LETX-VI under the physiological conditions is favorable for dopamine synthesis and has no obvious adverse influences on synaptic transmission. Generally, physiologically expressed α-synuclein is an essential presynaptic, activity-dependent negative regulator of synthesis and release of dopamine [53]. However, abnormal accumulation of α-synuclein has a central role in the pathogenesis of PD. Therefore, there is an urgent need to discover novel therapeutics that targets the protein α-synuclein [54]. Small-molecule chaperones (such as deoxycholic acid, ursodeoxycholic acid and tauroursodeoxycholic acid) presented the ability to prevent misfolding and aggregation of α-synuclein as well as to disentangle mature α-synuclein amyloid fibrils [55, 56]. However, due to toxicity constraints, these small molecules could not be translated into clinical drugs. How to develop novel drugs to prevent the toxic α-synuclein accumulation and thus reduce the risk of PD occurrence is an issue worthy of exploration.

Lewy bodies are a neuropathological feature of PD, and α-synuclein is considered the main component of Lewy bodies and the major biomarker of PD [57, 58]. Our present study demonstrated that the level of α-synuclein could be downregulated by LETX-VI, which implies that LETX-VI is able to inhibit the abnormal accumulation of α-synuclein and thus impedes the formation of Lewy bodies, preventing or delaying the development of PD. In order to confirm the in vivo efficency of LETX-VI inhibiting PD, we developed a PD mouse model with MPTP that is the most accepted gold standard neurotoxin widely utilized to induce the PD mouse model because it recapitulates most of the Parkinsonian symptoms in animal as like PD patients [59]. LETX-VI was shown to significantly ameliorate MPTP-induced behavioral impairments of the PD model mice.

In the nigrostriatal tissues of the MPTP-induced PD model mice, the level of α-synuclein was increased, whereas that of TH was decreased due to the adverse influence of excessive α-synuclein on dopaminergic neurons [47–49] and the TH degradation via the ubiquitin-proteasome system [60]. Therefore, in the MPTP-induced PD model mice significant loss of dopaminergic neurons in the substantia nigra was found and dopamine level was decreased by 50% or so [61, 62]. Our results were in agreement with the relevant reports in the cited literature, demonstrating that our development of PD mouse model was succesfully, although we did not quantitatively determinated the dopamine level in the nigrostriatal tissues. Application of LETX-VI almost completely abolished the toxic effects of MPTP (Fig. 7). TH immunoflurescence and Nissl staining demonstrated that LETX-VI could protect the neurons including dopaminergic neurons from the injury caused by MPTP-induced excessive α-synuclein. All the in vitro and in vivo results demonstrate that LETX-VI promotes the synthesis and secretion of dopamine via multiple mechanisms including preventing abnormal accumulation of α-synuclein, increases the level of dopamine and shows implications in the prevention and treatment of PD via endogenous pathways. Nevertheless, although we experimentally demonstrated that LETX-VI can downregulate the expression of α-synuclein and improve the behaviors of Parkinson’s disease, thus having potential application prospects for the prevention and treatment of PD, the relevant mechanism of action of LETX-VI is not completely clear and needs further investigation in the future work.

Dopamine can not cross the blood-brain carrier (BBB) (or has a poor BBB penetration ability), and therefore L-dopa is often orally administrated in the treatment of PD in clinic. After the L-dopa crosses BBB to enter the brain, it is converted into dopamine by the L-dopa decarboxylase (aromatic amino acid decarboxylase) in CNS, thus increasing the level of dopamine in the CNS tissues. Due to fact that L-dopa decarboxylase is rich in the periphery, L-dopa is usual administrated in combination with benserazide or carbidopa [9, 63]. Considering that L-dopa/benserazide formulation is a commonly used and most effective treatments for PD, in the present in vivo experiments, LETX-VI was administrated in combination with benserazide to even better simulate the treatment of PD in clinic [64, 65]. The role that benserazide plays is to reduce the conversion of the endogenous L-dopa produced in peripheral tissues to dopamine. The influence of benserazide on the activity of L-dopa decarboxylase in CNS tissues, if any, is small. The observed effects of LETX-VI/benserazide on CNS tissues are mainly attributed to the action of LETX-VI, rather than to that of benserazide.

## Conclusions

LETX-VI treatment of PC12 cells increased the total level of dopamine of PC12 cells and promoted the transport and release of the dopamine by upregulating the levels of TH and L-dopa decarboxylase, reducing that of monoamine oxidase B, and modulating the abundance and/or posttranslational modification of VMAT2 and DAT. At the same time, LETX-VI decreased the level of α-synuclein and mitigated the toxic effect of excessive α-synuclein on dopaminergic neurons. When applied to the MPTP-induced PD model mice, LETX-VI improved parkinsonian behaviors of the mice, mitigated MPTP-induced α-synuclein upregulation and TH downregulation, and displayed protective effects on neurons particularly dopaminergic neurons. Both in vitro and in vivo observations have consistently demonstrated that LETX-VI possesses the potential in the prevention and treatment of PD.

## Methods

### Culture and LETX-VI treatment of PC12 cells

PC12 cell line was purchased from the Cell Bank of Type Culture Collection of the Chinese Academy of Sciences (Shanghai, China). The PC12 cells were passaged in Dulbecco’s modified Eagle’s medium (DMEM) supplemented with 10% FBS and 100-unit penicillin-streptomycin of a series of 10-cm culture dishes, which were then incubated in an incubator at 37 ^o^C with 5% CO_2_ and 95% humidity. The culture dishes were randomly grouped into control and treatment groups. When the cells were grown to 80–90% confluence, the culture medium was aspirated and the cells were washed twice with PBS. The lyophilized recombinant LETX-VI, prepared by heterologous expression in *E.coli* according to the method previously described [66], was dissolved in serum-free DMEM to desired final concentrations, which were added into the dishes in the treatment group. The control cells were cultured with equal volume of serum-free DMEM. After 24 h, the PC12 cells were harvested for western blotting and other analyses.

### Western blot analysis

The protein samples prepared by homogenizing the PC12 cells of control, model and treatment groups in a PBS buffer were resolved on a 10% SDS-PAGE gel based on the method as described by Laemmli [67]. After the completion of electrophoresis, the gel block containing the protein of interest was cut off and the protein was transferred onto a PVDF membrane (Millipore, MA, USA) with a blot electrotransfer apparatus in the wet transfer method (200 mA/1.5 h). The membrane was blocked in 5% milk/TBST (50 mM Tris-HCl, 150 mM NaCl, 0.1% Tween-20, pH 7.5) for 3.0 h at room temperature. The primary antibody diluted with the TBST buffer was added and incubated on a shaker at 4 ^o^C overnight. After the membrane was washed three times with the TBST buffer for 10 min each, the secondary antibody that was diluted with TBST buffer was added and incubated at room temperature for 1 h. Then the membrane was washed three times with TBST buffer for 10 min each. The blot was developed using the enhanced chemiluminescence (ECL) method (Thermo Scientific, USA) and recorded with a ChemiDoc XRS imaging system (Bio-Rad, USA).

Antibodies for α-synuclein and GAPDH were purchased from Abcam (Cambridge, UK), those for TH, DAT, VMAT2 and actin from Proteintech (Wuhan, China), and those for monoamine oxidase A, monoamine oxidase B, catechol-O-methyltransferase, junction plakoglobin and 6 ×His tag from CUSABIO (Wuhan, China).

### Determination of dopamine

Quantitative determination of dopamine was carried out by referring to the fluorometric method described by Schlumpf et al. [68]. For establishing a standard curve of dopamine, a series of small eppendorf tubes containing different concentrations (0, 40, 80, 120, 160 and 200 ng) of standard dopamine in 300 µl aqueous solution were prepared, and 200 µl 0.2 M PBS buffer (pH 6.9) was added to each tube. The dopamine in the tubes was oxidized for 3 min by adding 200 µl iodine solution (0.1 M in ethanol). After the the oxidative reaction was stopped by addition of 200 µl Na_2_SO_3_ (0.5 g Na_2_SO_3_ in 2 ml H_2_O + 18 ml 5 M NaOH), 100 µl glacial acetic acid was added 2 min later, followed by heating at 100 ^o^C for 6 min and, after cooling to room temperature, centrifugation at 16 900 g for 5 min. The supernatant was collected and tested with a fluorescence spectrophotometer (Model F97, Lengguang Tech, China) at excitation/emission maxima of 324/375 nm. The fluorometric quantification of dopamine in culture medium and PC12 cells was performed basically as the same as above. After the specified treatment period, the culture medium containing PC12 cells was collected and then separated by low-speed centrifugation. The separated culture medium and the cells were used to determine the extracellular and intracellular dopamine, respectively. For measuring the intracellular dopamine content, the isolated cells were lysed by treatment with freeze/thaw cycles and then centrifuged at about 15 000 g for 5 min. The resulting supernatant was collected for the determination of the dopamine within PC12 cells.

### Overexpression of α-synuclein

The *SNCA* gene sequence encoding rat α-synuclein was obtained from NCBI and chemically synthesized. The sequence was amplified by PCR and then ligated into eukaryotic expressing vector pcDNA3.1(+). The constructed recombinant plasmids pcDNA3.1 (+)-*SNCA* were identified by double enzyme digestion using BamHI and EcoRI, followed by agar gel electrophoresis. The recombinant plasmids were transfected into TOP10 for colony sequencing. Before the constructed expression plasmids were transfected into PC12 cells with Lip2000 for α-synuclein overexpression, the transfection efficiencies of different Lip2000 to plasmid ratios were compared in order to screen the optimal transfection conditions.

### Real-time fluorenscent quantitative PCR (qPCR)

To validate the effect of LETX-VI on the expression of the TH at the transcriptional level, real-time fluorenscent quantitative PCR (qPCR) was employed to analyze the mRNA level of the TH in the PC12 cells with or without LETX-VI treatment. Briefly, PC12 cells were seeded in DMEM supplemented with 10% FBS and 100-unit penicillin-streptomycin of six 10-cm culture dishes, which were randomly grouped into control and treatment groups, each consisting of three dishes. The culture dishes were then cultured in an incubator at 37 ^o^C with 5% CO_2_ and 95% humidity. When the cells were grown to 80–90% confluence, the culture medium was removed by aspiration and the cells were washed twice with PBS. LETX-VI was dissolved in serum-free DMEM to 1 µM, which were added into the culture dishes in the treatment group. Equal volume of serum-free DMEM was added into the control dishes. After being cultured for 24 h, the PC12 cells were harvested for subsequent analyses. For obtaining total RNA, Trizol-chloroform extraction was performed, followed by isopropanol precipitation. The cDNA was synthesized and then used as the template for PCR amplification of the TH with the following primers: Forward primer for TH, TGTCACGTCCCCAAGGTTCA; Reverse primer, GAGAACAGCATTCCCATCCCT; Forward primer for GAPDH, ACAGCAACAGGGTGGTGGAC; Reverse primer, TTTGAGGGTGCAGCGAACTT. PCR amplification was performed under the following conditions: preincubation for 10 min at 95 ^o^C, followed by 40 cycles of denaturation for 15 s at 95 ^o^C, annealing and extension for 30 s at 60 ^o^C. All the samples were run at least in triplicates.

### HIS-LETX-VI pulldown

In order to prepare HIS-tagged LETX-VI (HIS-LETX-VI), the LETX-VI gene was amplified by PCR and was cloned into the expression vector pET32a. HIS-tagged LETX-VI fusion protein was expressed using *E. coli* BL21 (DE3) by IPTG induction. The expressed fusion protein was affinity purified with Ni-NTA beads. For preparing the PC12 cell lysate, PC12 cells were seeded in 10-cm culture dishes containing DMEM supplemented with 10% FBS. The cells were cultured in a 37 ^o^C incubator containing 5% CO_2_ and humidified air until the PC12 cells were grown to approximately 80–90% confluence. The culture medium was sucked away and the PC12 cells were washed twice with PBS, followed by cell collection and lysis in a lysis buffer (50 mM Tris, 150 mM NaCl, 1% Triton X-100, protease and phosphatase inhibitor cocktai, 2 mM DTT, pH 8.0) with sonication. After centrafugation at 16 900 g for 15 min at 4 ^o^C, the supernatant was collected and precleared by incubation with Ni-NTA bead-bound HIS tag protein for 8–10 h at 4 ^o^C under slow continuous rotation. For HIS-LETX-VI pulldown, the precleared cell lysate was incubated with Ni-NTA bead-bound HIS-LETX-VI under the same conditions. Then the Ni-NTA beads were collected by centrifugation and washed three times with a washing buffer (50 mM Tris, 150 mM NaCl, 1% Triton X-100, 2 mM DTT, pH 7.8). Finally, a loading buffer was added, followed by boiling for 10 min and centrifugation. The supernatant was collected for western blot analysis.

### Immunofluorescence analysis of DAT in PC12 cells

In order to prepare cell-attached slides, the cover slides were cleaned by repeated wshing, dried in the flame of an alcohol lamp and placed in a 6-well plate, followed addition of 100 µl polylysine in each well. After incubation at 37 ^o^C for 2 h, each well was washed three times with PBS buffer. PC12 cells were inoculated in each well and incubated in an incubator containing 5% CO_2_ at 37 ^o^C. When the cells were grown to 75–85% confluence, the PC12 cells were treated with LETX-VI for 24 h. Then the cells were washed three times with PBS and then fixed with 4% paraformaldehyde for 10 min, followed by washing three times with PBS. The cells were treated with PBS containing 0.5% Triton X-100 for 3 min and washed again with PBS. For antibody incubation, the PC12 cells were blocked in 5% goat serum for 1 h at room temperature. The primary DAT antibody diluted with 5% goat serum was added and incubated overnight at 4 °C. After the cells were washed three times with PBS for 5 min each, the diluted green fluorescent-labeled secondary antibody solution was added and incubated at room temperature for 1 h. Then the cells were washed three times with PBS for 5 min each. For nuclear staining, the PC12 cells were treated with DAPI (4′, 6-diamidino-2-phenylindole) for 15 min and then washed again with PBS. The coverslips were mounted on microscope slides. The fluorescence images were captured on a fluorescence microscope (Zeiss Axio Imager M2, German). Fluorescence intensity analysis was performed using the ImageJ software.

### Development of PD mouse model and LETX-VI treatment

Male C57BL/6J mice (18–20 g each) were maintained in a 12 h/12 h light/dark cycle, under ambient temperature of 25 ± 1 °C and relative humidity of 55 ± 10%, allowed free access to food and water. All the mice were acclimatized for 1 week before being used in subsequent experiments. The mice with limb dyskinesia were excluded. A subacute mouse model of PD was established according to the previous method [69, 70]. Briefly, male C57BL/6J mice were randomly divided into three groups: control group, model group and MPTP + LETX-VI treatment group (treatment group for short), with 5 mice in each group. The mice in control group were intraperitoneally injected with sterilized physiological saline. Model group mice were intraperitoneally injected with MPTP in sterilized physiological saline at a dose of 30 mg/kg body weight daily for 6 continuous days. The mice in treatment group were first intraperitoneally injected with MPTP at a dose of 30 mg/kg body weight and 30 min later injected with LETX-VI at a dose of 10 mg/kg body weight and benserazide at a dose of 6.25 mg/kg body weight daily to inhibit peripheral L-dopa decarboxylase [71] for 6 continuous days. The injection volume was 200 µl. After completion of the intraperitoneal injections, behavioral assessment and in vivo biochemical as well as histological analyses were performed.

The study was performed in accordance with the recommendations of the Guide for the Care and Use of Laboratory Animals of the China National Institute of Health and the Ethics Committees of Hunan Normal University ratified all the experiments with animals.

### Behavioral tests

The performed behavioral experiments included pole test, wire-hanging test and footprint test. Pole test was carried out based on the previous method [72]. A wooded rod (1 cm in diameter, 50 cm in length) was vertically placed and a ball (5 cm in diameter) was fixed at the top. The rod was wrapped in gauze to increase the friction of the mouse climbing. The mouse was positioned on the pole by sliding the forepaws over the ball while holding the animal by tail. The total time required to climb down the pole were measured. After completion of the 6-day drug administration, each mouse was daily tested in 4 successive trials, with a 30-min interval during which 75% ethanol was used for odor clearance.

Wire-hanging test was made according to the previous method with some modifications [73, 74]. In the wire-hanging test, the mouse hung by its forelimbs from a horizontal iron wire (1 mm in diameter) stretched between two posts about 30 cm above the ground. The time when the animal fell was recorded. The results were assessed by a 4-grade scoring system: during 20 s, 0 point for failing from the wire, 1 point for remaining on the wire with obvious tremor and difficulty in maintaining balance, 3 points for remaining on the wire without movement and difficulty in maintaining balance, 5 points for freely and stably moving on the wire. The test was daily repeated four times with a 30-min interval for 4 continuous days after completion of the 6-day drug administration.

Footprint test was conducted as previously described [75]. For footprint test, the hind paws of the mouse were painted with black ink and the mouse was trained to walk through a homemade wooden trough (80 × 5 × 5 cm) with a white paper placed at the bottom. The stride lengths were measured and analyzed only when the mouse ran at a constant velocity. Strides over the first and last 5 cm of the passage were excluded because of the variation in the walking velocity of the mouse. The test was daily repeated 4 times with a 30-min interval for 3 continuous days after completion of the 6-day drug administration.

### Biochemical analysis of brain tissues

After the behavioral tests were completed, the mice were sacrificed by cervical dislocation to make sure minimal pain [12]. The brain was quickly obtained on ice and frozen in liquid nitrogen. For performing western blot analysis, the brain was dissected and the tissues containing nigrostriatal region were taken and homogenized in the RIPA lysis buffer (Beyotime, Shanghai, China) with a mortar and pestle, followed by centrifugation at 14 000 g for 5 min at 4 ^o^C. The supernatant was collected for western blot analysis of the dopamine metabolism- and PD-related proteins in the same method as described earlier.

### Tyrosine hydroxylase immunofluorescence and Nissl staining

TH immunofluorescence staining was performed according to the methods previously described, with some modifications [76, 77]. The brain was dissected on ice and the tissues containing nigrostriatal region was collected and fixed with 4% paraformaldehyde for 24 h at 4 ^o^C. The sampled brain tissues were washed three times with PBS buffer (pH 7.4) and put into a dehydration box, followed by gradient alcohol dehydration with 75–100% alcohol and treatment with alcohol benzene, dimethylbenzene and 65 ^o^C melted paraffin. Then the brain tissues were embedded into paraffin and coronal sections were cut through the nigrostriatal tissue region using a paraffin slicer (LEICA RM2135, Germany). For TH immunofluorescence analysis, the paraffin sections were dewaxed in dimethylbenzene, rehydrated through treatment with decreasing concentrations of ethanol, and washed in distilled H_2_O, followed by antigen repairing by microwave heating and blockage in 3% BSA. The sections were incubated with anti TH antibody diluted in PBS buffer at 4 ^o^C overnight. After washing three times with a PBS buffer, the sections were incubated with a Cy3-conjugated secondary antibody at room temperature for 50 min in dark. The nuclei were stained with 2-(4-Amidinophenyl)-6-indolecarbamidine dihydrochloride (DAPI). Immunofluorescent images were acquired using a fluorescence microscope (Nikon Eclipse C1, Japan). The excitation/emission maxima for DAPI were 330–380 nm/420 nm, and those for Cy3 were 510–560 nm/590 nm.

Nissl staining was performed by referring to the described method with some modifications [78, 79]. For Nissl staining, the paraffin sections were sequentially treated with dimethylbenzene, anhydrous ethanol, 75% ethanol and distilled water. Then the sections were stained in toluidine blue for 5 min, followed by washing with distilled water and differentiation treatment with 1% glacial acetate. After rinsing with distilled water, the sections were dried in an oven and treated with dimethylbenzene for 10 min. Finally, the sections were sealed with neutral gum and observed under a light microscope (Nikon EclipseE100, Japan).

### Statistical analysis

All the experiments were performed at least in triplicate. The means and standard deviations (SD) for the control, model and treatment groups were calculated. The data are presented as mean ± SD. One-way ANOVA with Tukey’s post hoc test was used to analyze differences among three or more groups, and the paired *t*-test was used for statistical analysis of differences between 2 groups. The differences were considered significant at *P* < 0.05.

### Electronic supplementary material

Below is the link to the electronic supplementary material.

Supplementary Material 1. Additional file 1: Identification of α-synuclein expression vector and screening of optimal transfection conditions.

Supplementary Material 2. Additional file 2: Supplementary original gel electrophoretic and WB Figures.

Supplementary Material 3. Additonal file 3: Supplementary 4× and 10× immunofluorescence and Nissl staining images.

Supplementary Material 4

## Data Availability

All data are available in the manuscript and supplementary information.

## Abbreviations

CNS: Central nervous system
COMT: Catechol-O-methyltransferase
DAPI: 4',6-Diamidino-2-phenylindole
DAT: Dopamine transporter
Jup: Junction plakoglobin
LETX-VI: Latroeggtoxin-VI
MAO A: Monoamine oxidase A
MAO B: Monoamine oxidase B
MPTP: 1-Methyl-4-phenyl-1,2,3,6-tetrahydropyridine
Nurr 1: Nuclear receptor related protein 1
PD: Parkinson’s disease
α-Syn: α-Synuclein
TH: Tyrosine hydroxylase
VMAT2: Vesicular monoamine transporter 2
