# Supplementary material for: Effects of latroeggtoxin-VI on dopamine and α-synuclein in PC12 cells and the implications for Parkinson’s disease
Source: Biol Res. 2024 Mar 16;57:9. doi: 10.1186/s40659-024-00489-y (PMC10943915; doi:10.1186/s40659-024-00489-y)
Supplement: Supplementary file 1 — Supplementary Material 1. Additional file 1: Identification of α-synuclein expression vector and screening of optimal transfection conditions. [file 40659_2024_489_MOESM1_ESM.pdf]

湖南师范大学生物医学研究伦理委员会伦理审批件

伦审科2019第（231）号

项目名称	间斑寇蛛卵粒毒素-VI 影响 PC12 细胞多巴胺代谢与释放的分子机制研究				
承担专业	生物化学与分子生物学	承担责任	☑负责 ☐参与	申请人	王贤纯
研究分类*	1☑ 2 ☐ 3☑		研究起止时间	2019.1.1--2022.12.31	
研究类型	☑国家自然科学基金项目 ☐其它				
所在单位	生命科学学院				
课题来源	☑政府 ☐基金会 ☐公司 ☐国际组织 ☐其他				

递交审查资料：☑项目申请书 ☐研究论文 ☐其它

联系电话：13667399130

研究主要内容：系统探究间斑寇蛛卵粒毒素-VI (Latroeggtoxin-VI, LETX-VI）影响 PC12 细胞多巴胺代谢与释放的分子机制及可能的应用前景。具体包括 LETX-VI 进入 PC12 细胞的过程与分子基础，影响多巴胺代谢，转运和释放的作用机理，从而探明 LETX-VI 影响神经细胞的分子机制，为研究突触传递调控机制积累新的实验资料，并为揭示 LETX-VI 在药理学研究和相关疾病的防治等方面的潜在意义奠定实验基础。研究涉及 PC12 细胞系（中国科学院细胞库）和部分小鼠。

审查结果

二级单位伦理分委员会或所在学院审查意见：

经审查，该研究的实验设计和方案充分考虑了安全性和公平性原则，充分考虑了进行实验时的替代、减少和优化三原则，保护动物的权益，并将最大程度减轻动物的疼痛、痛苦和紧张，实验材料获取方式正规、合法。研究内容和研究结果不存在利益冲突。

签章：[signature]

日期：2019.6.8

伦理委员会意见：

☑同意 ☐修改后同意 ☐修改后重审 ☐不同意

伦理委员会主任委员签章：[signature]

伦理委员会公章：[stamp: 湖南师范大学 伦理委员会]

日期：2019.6.10

*研究分类：1=涉及动物实验； 2=涉及人体实验； 3=涉及组织或细胞实验
