# Supplementary material for: Effects of latroeggtoxin-VI on dopamine and α-synuclein in PC12 cells and the implications for Parkinson’s disease
Source: Biol Res. 2024 Mar 16;57:9. doi: 10.1186/s40659-024-00489-y (PMC10943915; doi:10.1186/s40659-024-00489-y)
Supplement: Supplementary file 2 — Supplementary Material 2. Additional file 2: Supplementary original gel electrophoretic and WB Figures. [file 40659_2024_489_MOESM2_ESM.pdf]

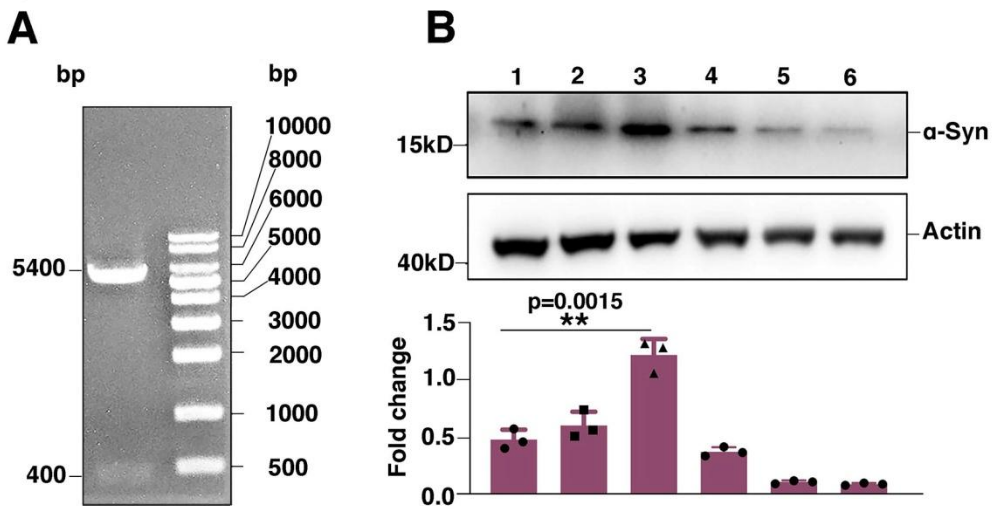

Additional File 1 Identification of α-synuclein expression vector and screening of optimal transfection conditions

A Gel electrophoretic identification of α-synuclein expression vector after double restriction enzyme digestion. **B** Western blot analysis of α-synuclein expression level to screen the optimal Lipofectamin 2000 to plasmid pcDNA 3.1 (+) - *SNCA* ratio. 1: control. 2: lipofectamin 2000. 3: 3 μl lipofectamin 2000 and 3 μg plasmid. 4: 6 μl lipofectamin 2000 and 3 μg plasmid. 5: 7 μl lipofectamin 2000 and 3 μg plasmid. 6: 9 μl lipofectamin 2000 and 3 μg plasmid.
