# Supplementary material for: Effects of latroeggtoxin-VI on dopamine and α-synuclein in PC12 cells and the implications for Parkinson’s disease
Source: Biol Res. 2024 Mar 16;57:9. doi: 10.1186/s40659-024-00489-y (PMC10943915; doi:10.1186/s40659-024-00489-y)

Supplemenetary original images of the representative western blot and gel electrophoretic images shown in the manuscript and the Additional file 1

1. Original WB images of the WB images in Fig. 1B

α-Syn:

Actin:

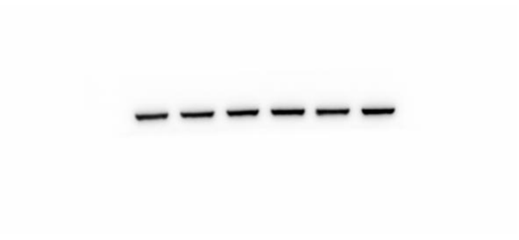

2. Original gel electrophoretic and WB images for the images in Additional file 1 and Fig. 2

2.1 Original gel electrophoretic image for the image in Additional file 1A

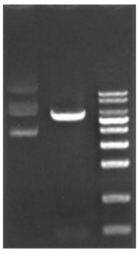

2.2 Original WB images for the WB images in Additional file 1B

α-Syn: （overexpression）

Samples loaded in left image：

Lane 1：Control；Lane 2：NC；Lane 3：α-Syn (+)

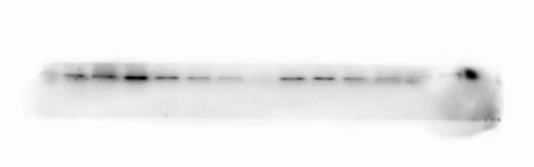

Actin:

Samples loaded in left image：

Lane 1：Control；Lane 2：NC；Lane 3：α-Syn (+)

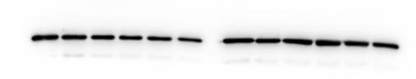

3. Original WB images for the WB images in Fig. 2

α-Syn (overexpression):

Samples loaded in left image：

Lane 1：Control；Lane 2：NC；Lane 3：α-Syn (+)

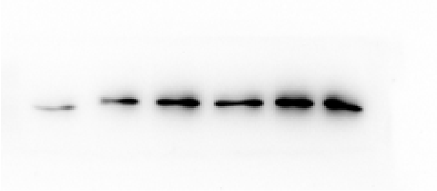

Actin:

Samples loaded in left image：

Lane 1：Control；Lane 2：NC；Lane 3：α-Syn (+)

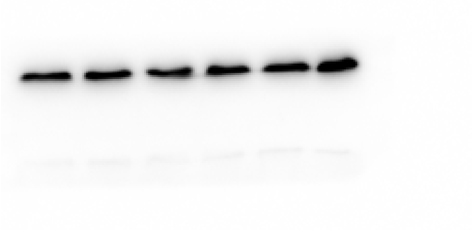

4. Original WB images for the WB images in Fig. 3

4.1 Original WB images for the WB images in Fig. 3A

TH:

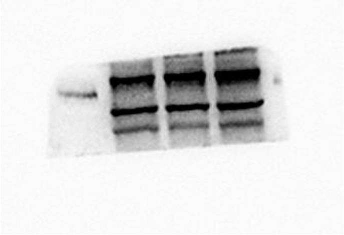

GAPDH:

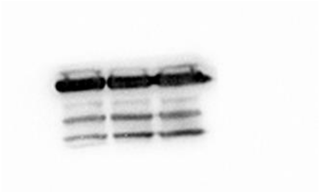

4.2 Original WB images for the WB images in Fig. 3B

p-TH:

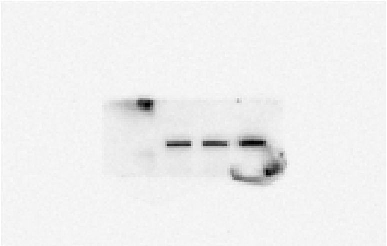

GAPDH:

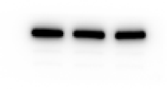

4.3 Original WB images for the WB images in Fig. 3D

Jup:

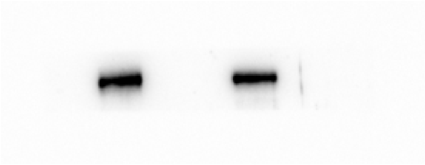

Nurr 1:

Samples loaded in left image：

Lane 1：Input；Lane 2：Vector；Lane 3：pull-down

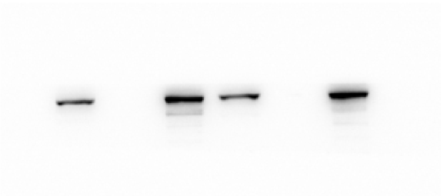

4.4 Original WB images for the WB images in Fig. 3E

Jup:

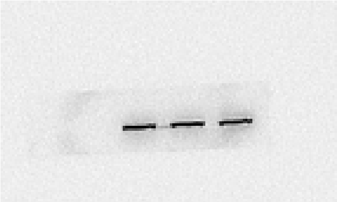

GAPDH:

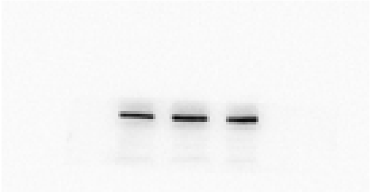

4.5 Original WB images for the WB images in Fig. 3F

Nurr 1:

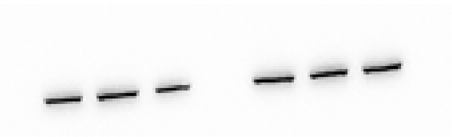

GAPDH:

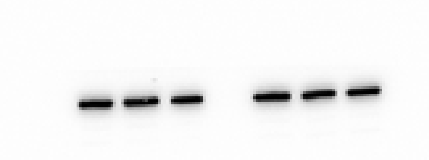

5. Original WB images for the WB images in Fig. 4

5.1 Original WB images for the WB images in Fig. 4A

DDC:

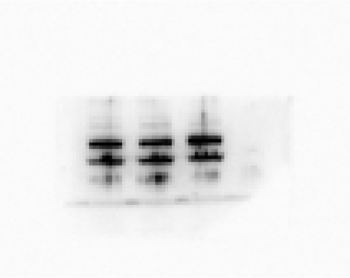

GAPDH:

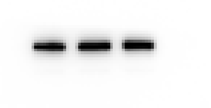

5.2 Original WB images for the WB images in Fig. 4B

MAO A:

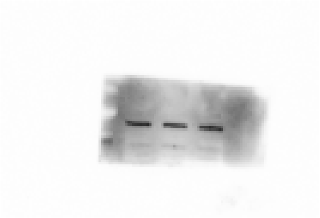

GAPDH:

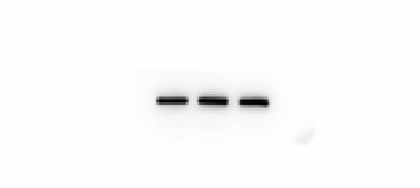

5.3 Original WB images for the WB images in Fig. 4C

MAO B:

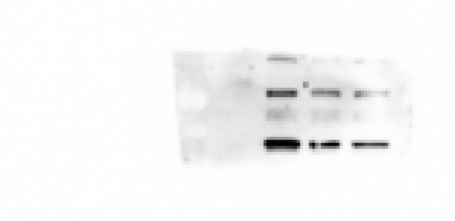

GAPDH:

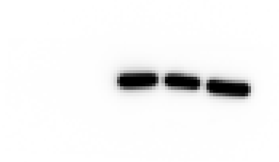

5.4 Original WB images for the WB images in Fig. 4D

COMT:

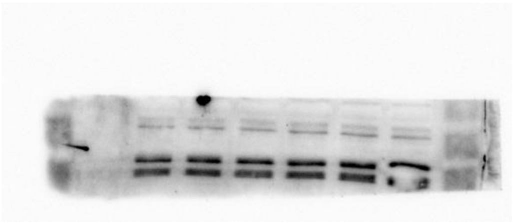

ACTIN:

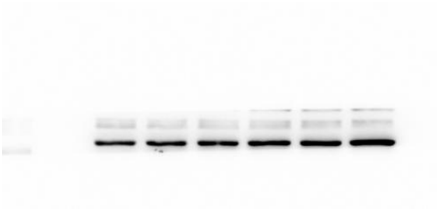

6. Original WB images for the WB images in Fig. 5

6.1 Original WB images for the WB images in Fig. 5A

VMAT 2:

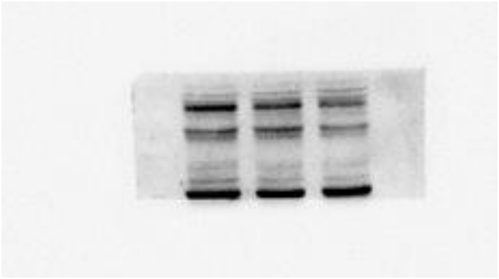

GAPDH:

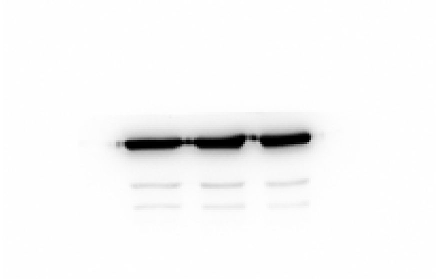

6.2 Original WB images for the WB images in Fig. 5B

DAT:

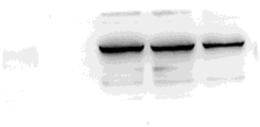

GAPDH:

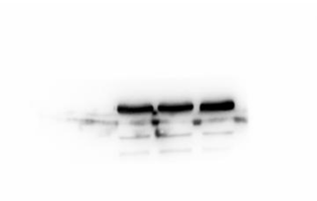

6.3 Original WB images for the WB images in Fig. 5C

p-DAT:

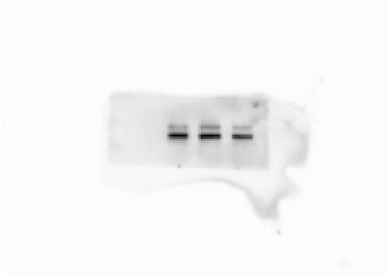

GAPDH:

7. Original WB images for the WB images in Fig. 7

7.1 Original WB images for the WB images in Fig. 7A

TH:

Samples loaded in left image：

Lane 4：Control；Lane 5：MPTP；Lane 6：MPTP + LETX-VI

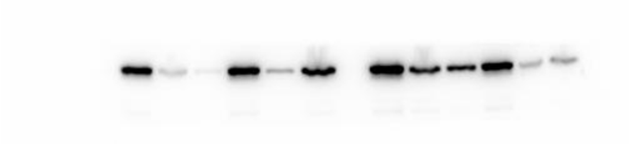

GAPDH:

Samples loaded in left image：

Lane 4：Control；Lane 5：MPTP；Lane 6：MPTP + LETX-VI

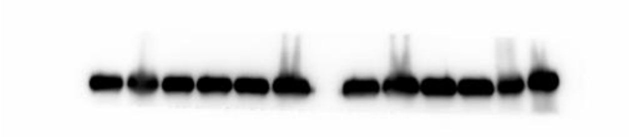

7.2 Original WB images for the WB images in Fig. 7B

MAO B:

Samples loaded in the penultimate set of images

Lane 7：Control；Lane 8：MPTP；Lane 9：MPTP + LETX-VI

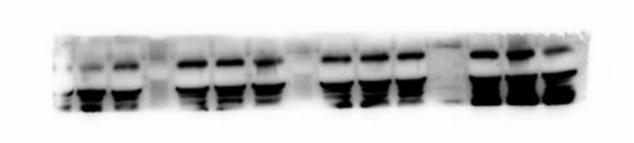

GAPDH:

Samples loaded in the middle set of images

Lane 7：Control；Lane 8：MPTP；Lane 9：MPTP + LETX-VI

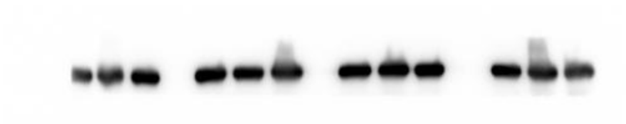

7.3 Original WB images for the WB images in Fig. 7C

COMT:

Samples loaded in the middle set of images

Lane 4：Control；Lane 5：MPTP；Lane 6：MPTP + LETX-VI

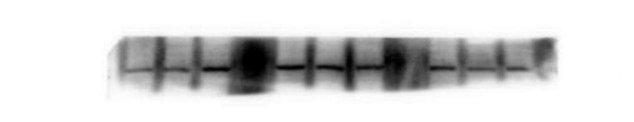

GAPDH:

Samples loaded in the middle set of images

Lane 4：Control；Lane 5：MPTP；Lane 6：MPTP + LETX-VI

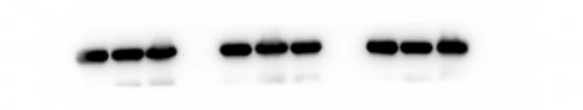

7.4 Original WB images for the WB images in Fig. 7D

DAT:

Samples loaded in the last set of images

Lane 7：Control；Lane 8：MPTP；Lane 9：MPTP + LETX-VI

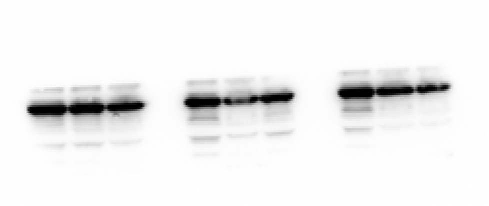

GAPDH:

Samples loaded in the last set of images

Lane 7：Control；Lane 8：MPTP；Lane 9：MPTP + LETX-VI

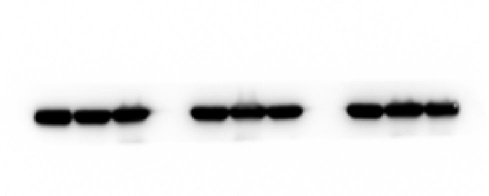

7.5 Original WB images for the WB images in Fig. 7E

α-Syn:

Samples loaded in the first set of images

Lane 1：Control；Lane2：MPTP；Lane 3：MPTP + LETX-VI

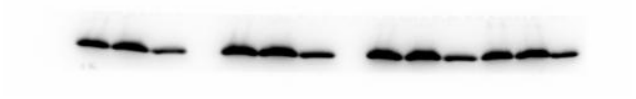

GAPDH:

Samples loaded in the first set of images

Lane 1：Control；Lane 2：MPTP；Lane 3：MPTP + LETX-VI

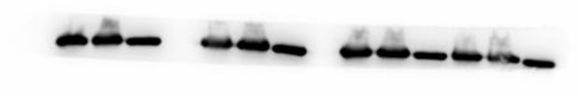

Supplement: Supplementary file 3 — Supplementary Material 3. Additonal file 3: Supplementary 4× and 10× immunofluorescence and Nissl staining images. [file 40659_2024_489_MOESM3_ESM.pdf]
