# Supplementary material for: Effects of latroeggtoxin-VI on dopamine and α-synuclein in PC12 cells and the implications for Parkinson’s disease
Source: Biol Res. 2024 Mar 16;57:9. doi: 10.1186/s40659-024-00489-y (PMC10943915; doi:10.1186/s40659-024-00489-y)

Supplementary 4× and 10× immunofluorescence and Nissl staining images

1. Immunofluorescence images for DAT analysis

Fig. 5D

4 ×

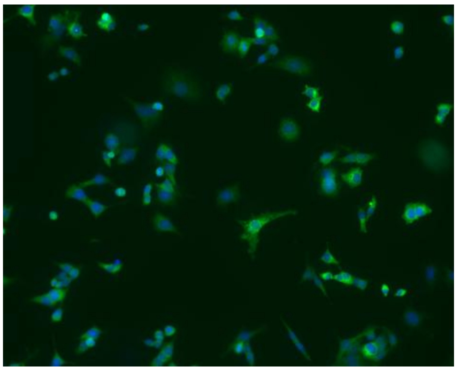

10 ×

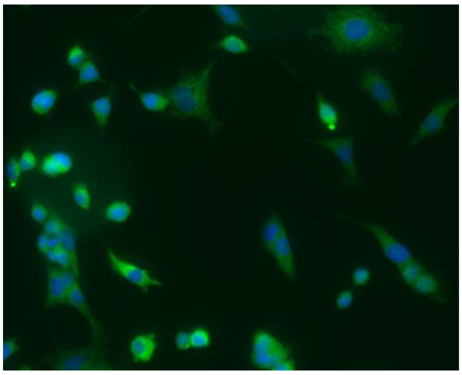

Fig. 5E

4×

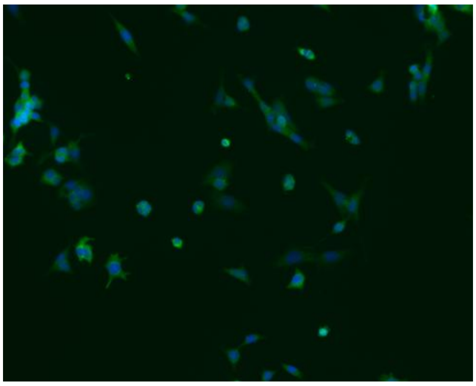

10 ×

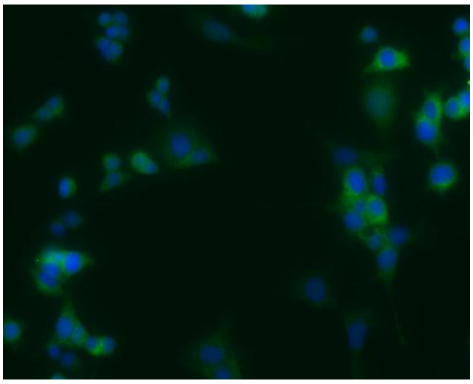

Fig. 5F

4×

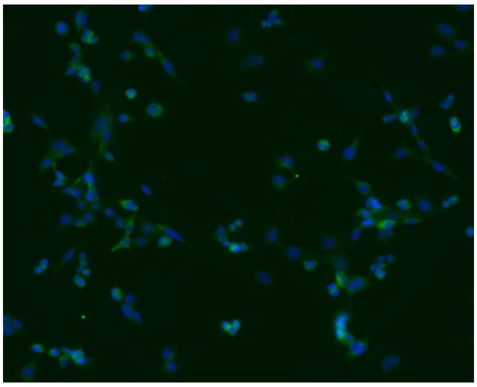

10 ×

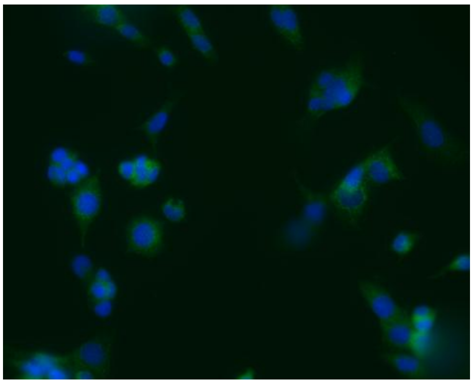

2. Immunofluorescence images for TH analysis

Fig. 8A

4 × images

Control:

DAPI

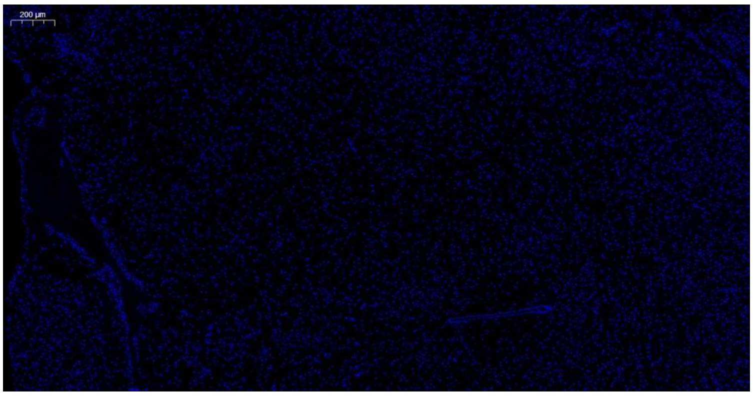

TH

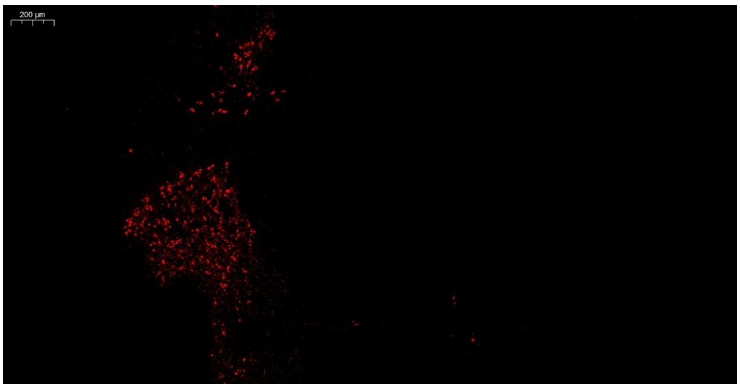

Merge

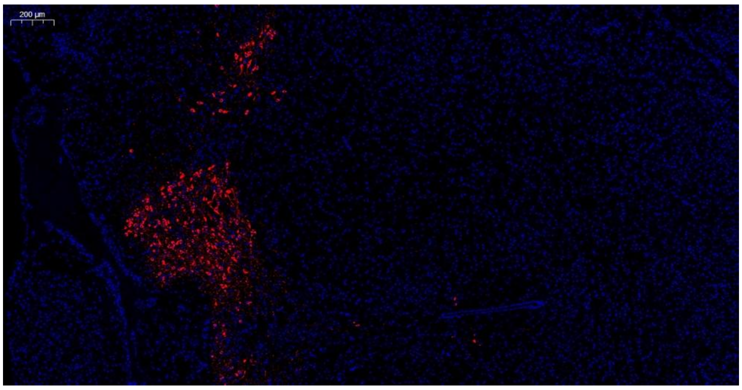

MPTP:

DAPI

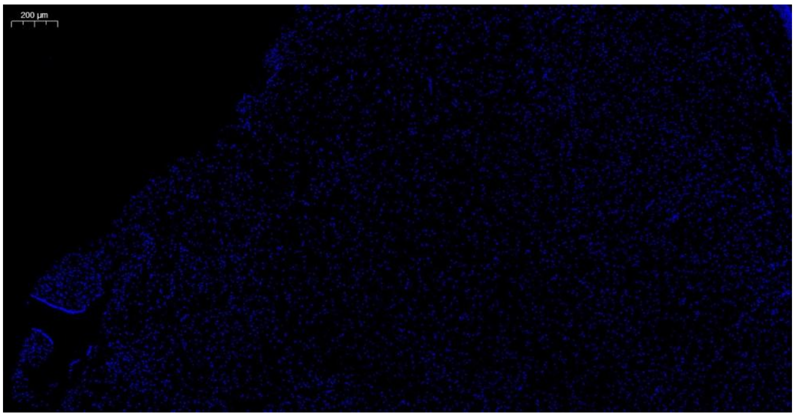

TH

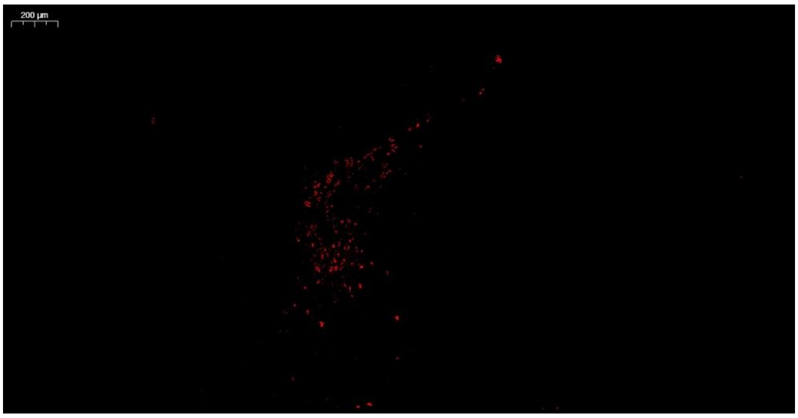

Merge

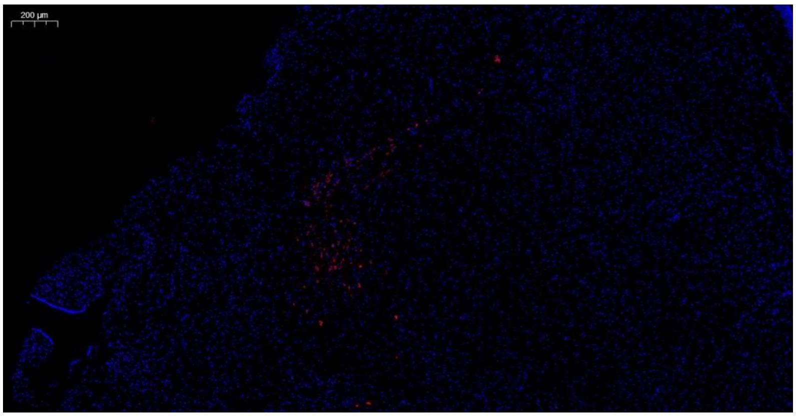

MPTP + LETX- I:

DAPI

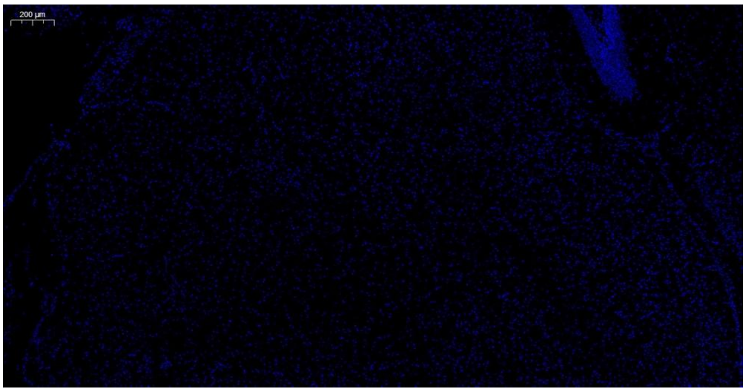

TH

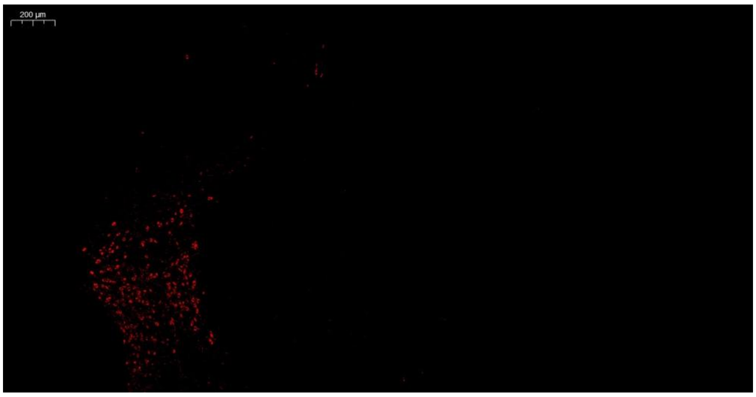

Merge

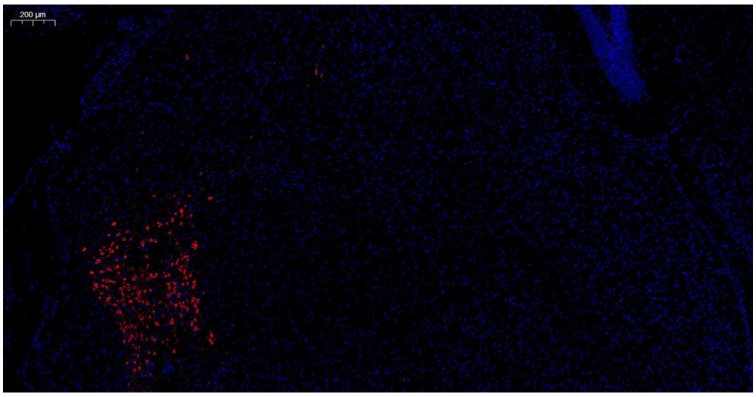

10 × images

Control:

DAPI

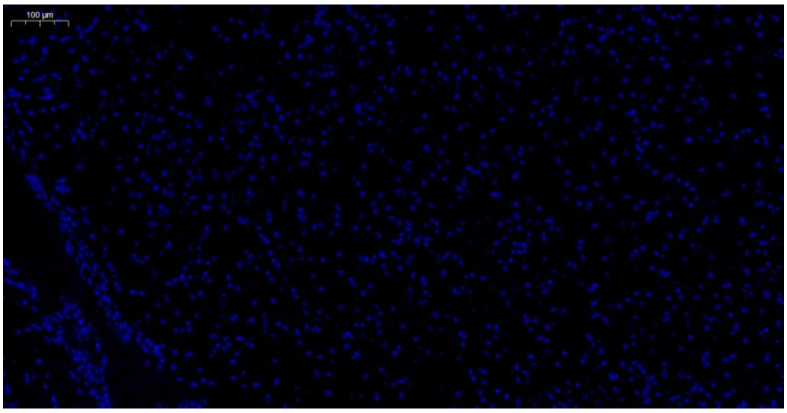

TH

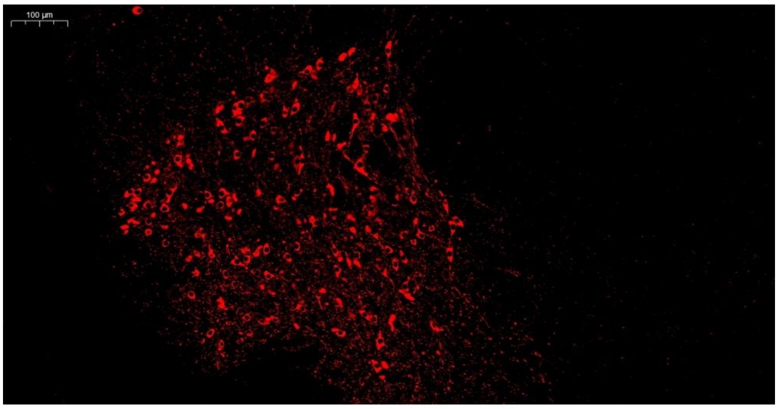

Merge

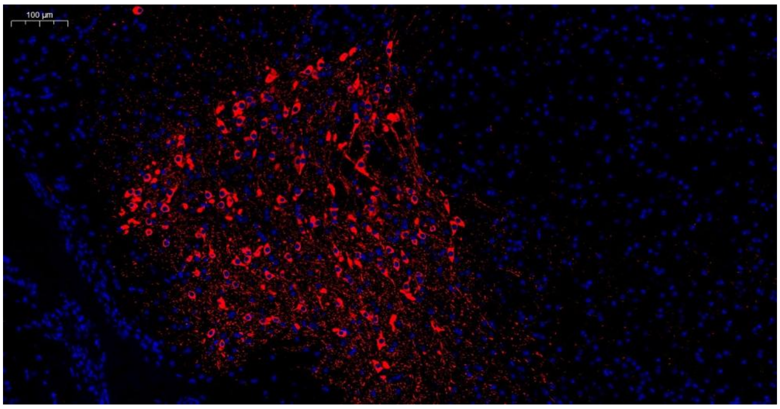

MPTP:

DAPI

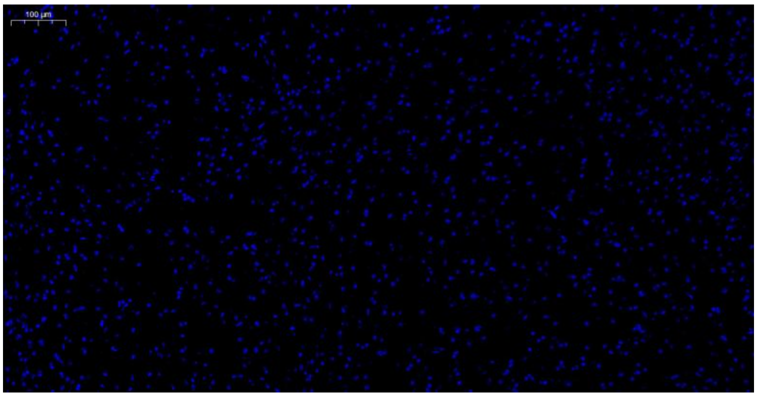

TH

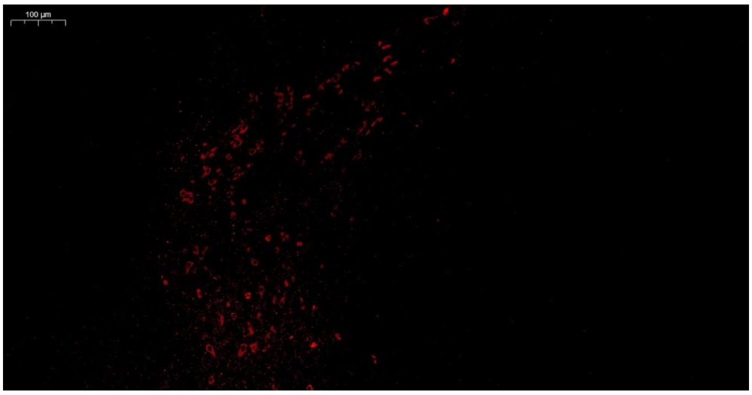

Merge

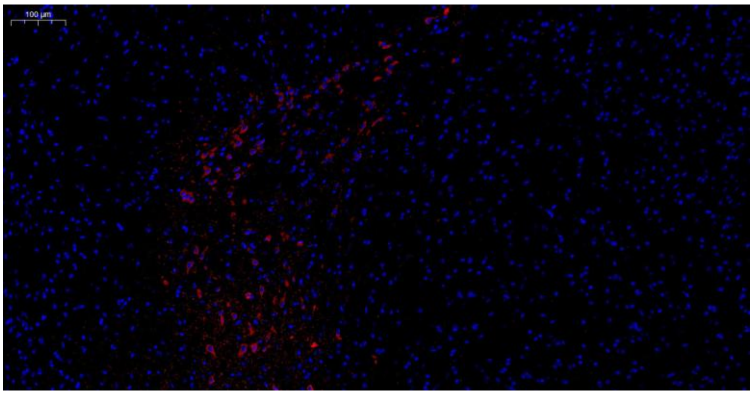

MPTP + LETX- VI:

DAPI

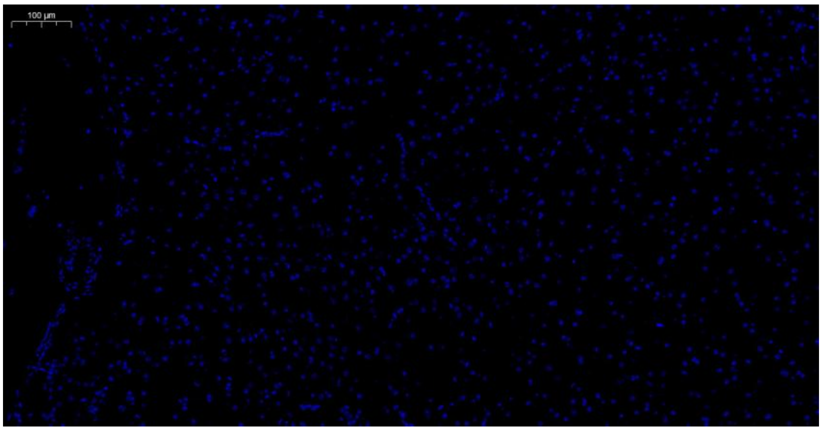

TH

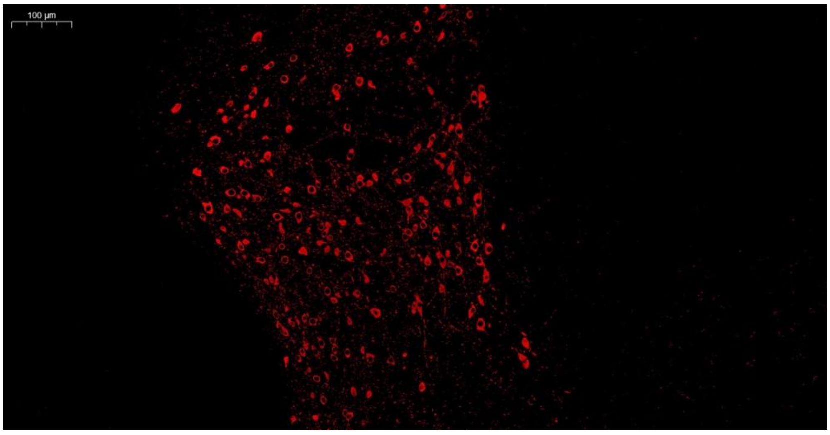

Merge

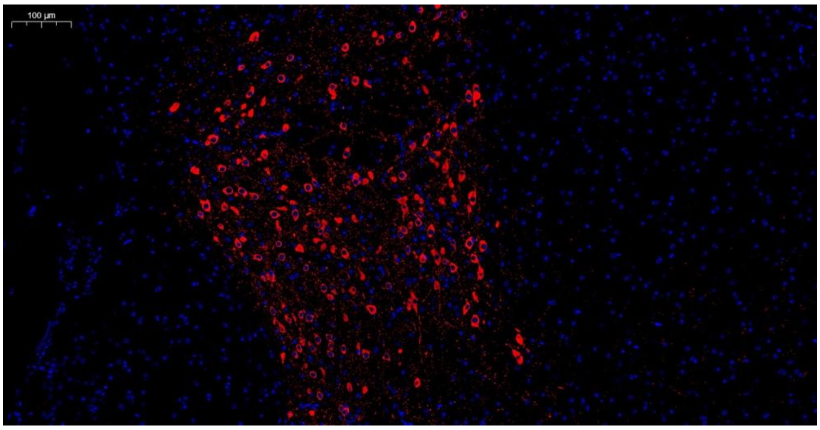

3. Nissl staining imagge for Fig. 8B

4 × images

Control

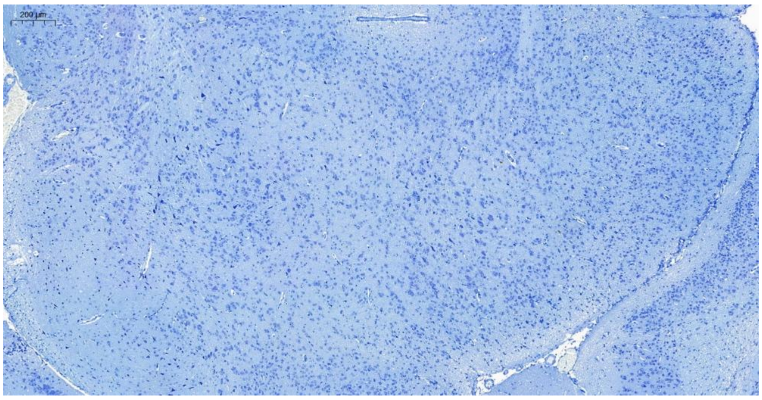

MPTP

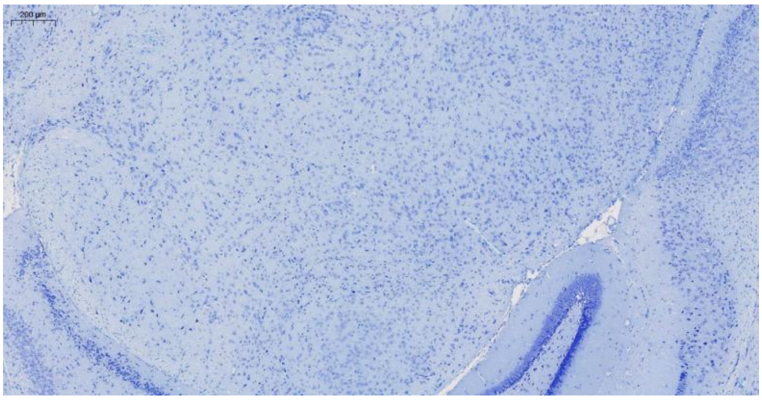

MPTP + LETX-VI:

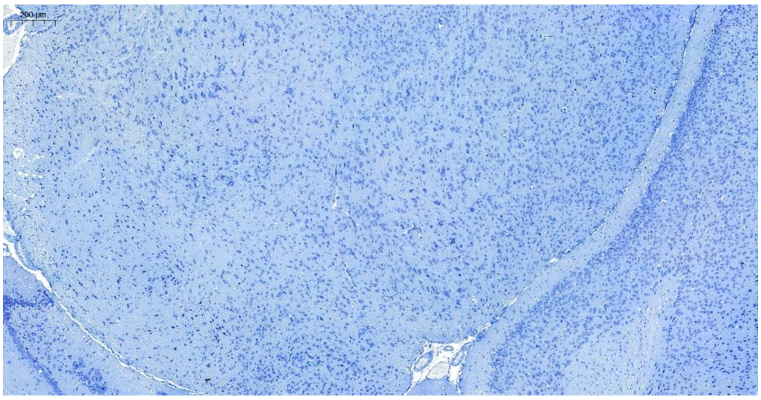

10 × images

Control

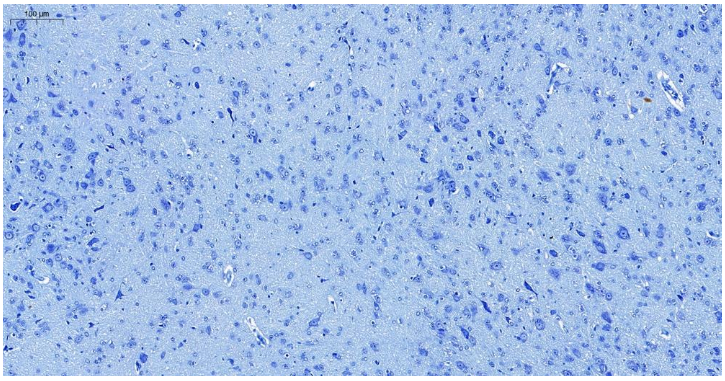

MPTP

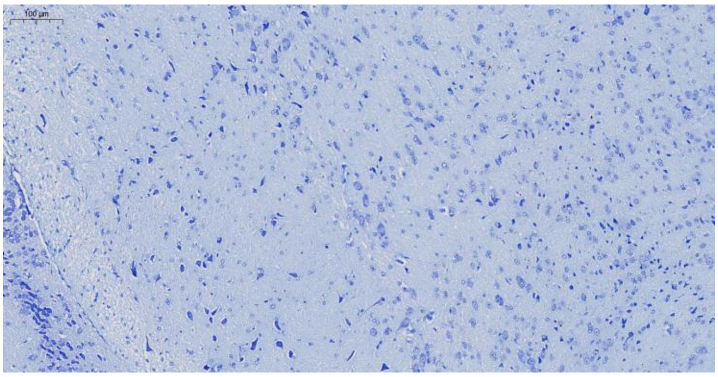

MPTP + LETX- VI

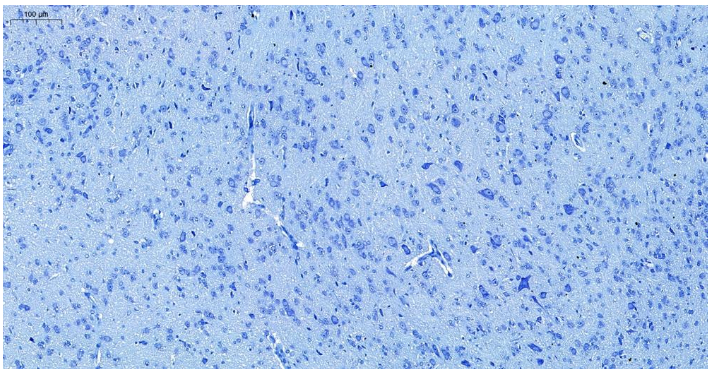

Supplement: Supplementary file 4 — Supplementary Material 4 [file 40659_2024_489_MOESM4_ESM.pdf]
